# Discovery of single nucleotide polymorphisms in candidate genes associated with fertility and production traits in Holstein cattle

**DOI:** 10.1186/1471-2156-14-49

**Published:** 2013-06-07

**Authors:** Sarah D Cochran, John B Cole, Daniel J Null, Peter J Hansen

**Affiliations:** 1 Department of Animal Sciences, D.H. Barron Reproductive and Perinatal Biology Research Program, and Genetics Institute, University of Florida, Gainesville, FL 32611-0910, USA; 2 Animal Improvement Programs Laboratory Agricultural Research Service, USDA, Beltsville, MD 20705-2350, USA

**Keywords:** Daughter pregnancy rate, Fertility, Dairy cattle, SNP, Candidate gene

## Abstract

**Background:**

Identification of single nucleotide polymorphisms (SNPs) for specific genes involved in reproduction might improve reliability of genomic estimates for these low-heritability traits. Semen from 550 Holstein bulls of high (≥ 1.7; n = 288) or low (≤ −2; n = 262) daughter pregnancy rate (DPR) was genotyped for 434 candidate SNPs using the Sequenom MassARRAY® system. Three types of SNPs were evaluated: SNPs previously reported to be associated with reproductive traits or physically close to genetic markers for reproduction, SNPs in genes that are well known to be involved in reproductive processes, and SNPs in genes that are differentially expressed between physiological conditions in a variety of tissues associated in reproductive function. Eleven reproduction and production traits were analyzed.

**Results:**

A total of 40 SNPs were associated (*P* < 0.05) with DPR. Among these were genes involved in the endocrine system, cell signaling, immune function and inhibition of apoptosis. A total of 10 genes were regulated by estradiol. In addition, 22 SNPs were associated with heifer conception rate, 33 with cow conception rate, 36 with productive life, 34 with net merit, 23 with milk yield, 19 with fat yield, 13 with fat percent, 19 with protein yield, 22 with protein percent, and 13 with somatic cell score. The allele substitution effect for SNPs associated with heifer conception rate, cow conception rate, productive life and net merit were in the same direction as for DPR. Allele substitution effects for several SNPs associated with production traits were in the opposite direction as DPR. Nonetheless, there were 29 SNPs associated with DPR that were not negatively associated with production traits.

**Conclusion:**

SNPs in a total of 40 genes associated with DPR were identified as well as SNPs for other traits. It might be feasible to include these SNPs into genomic tests of reproduction and other traits. The genes associated with DPR are likely to be important for understanding the physiology of reproduction. Given the large number of SNPs associated with DPR that were not negatively associated with production traits, it should be possible to select for DPR without compromising production.

## Background

There is a negative genetic correlation between milk yield and fertility in dairy cattle [1-3]. Partly as a result, the large improvement in milk yield over the last 40 years was accompanied by a decline in fertility [4-6]. Genetic selection for fertility is hampered by low heritability. For example, the heritability for daughter pregnancy rate (DPR), the fertility trait most widely measured in the United States, has been estimated at 0.04% [2]. Genetic estimates of fertility can be improved by genome-wide single nucleotide polymorphism (SNP) arrays. Utilization of the BovineSNP50 chip from Illumina (San Diego, CA, USA) improved reliability for DPR [7,8] but the low heritability and polygenic nature of the trait has meant that improvements in reliabilities achieved by incorporation of genomic information was less than for other traits. Thus, while the incorporation of information from the SNP50 chip increased reliability of DPR by 17% in Holsteins, this improvement was one of the lowest of the 12 traits examined [8].

One possible way to improve the accuracy of genomic estimates of fertility is to incorporate SNPs for specific genes involved in reproduction into SNP panels. The bovine genome contains over 20,000 genes, and over 14,000 of those do not contain a single SNP on the BovineSNP50 chip [9]. Incorporation of candidate gene SNPs into genomic tests for reproduction would allow selection of causative SNPs or SNPs physically more close to causative SNPs. Such an approach has been successful for improving ability to detect genomic associations with disease [10].

Many genes have been associated with reproduction in the dairy cow. Among these are SNPs related to *in vitro* fertilization or development, such as *STAT5A*[11], *FGF2*[12,13] and *PGR*[14]), DPR (*CAST*[15]), sire conception rate including *STAT5A*[16], *FGF2*[16], and *ITGB5*[17], calving interval (*GHR*[18]), superovulation response (*FSHR*[19]), twinning rate (*IGF1*[20]) and incidence of still birth (*NLRP9*[21] and *LEP*[22]). In beef cattle, SNPs related to reproductive function include those in *HSPA1A,* associated with calving rate [23], and *PAPPA2*, associated with calving interval [24].

The previously mentioned SNPs only represent a small portion of the genes involved in reproductive processes. Recent studies have revealed genes whose expression in tissues or cells of importance to reproduction vary with reproductive status; these genes are candidates for containing SNPs that impact fertility. For example, genes were identified that were differentially regulated in the brain of cows displaying strong estrus compared to those displaying weak estrus [25], in the endometrium of heifers which produced viable embryos compared to those which produced non-viable embryos [26], and in biopsies from embryos that resulted in live calves as compared to embryos that died following embryo transfer [27]. Genetic variants in the genes differentially expressed in the aforementioned studies and others may be responsible for differences in fertility among animals.

The goal of the current study was to identify SNPs in candidate genes affecting reproductive processes. The approach was to evaluate effectiveness of SNPs in candidate genes for explaining genetic variation in DPR. Three types of SNPs were evaluated: SNPs previously reported to be associated with reproductive traits of dairy or beef cattle or physically close to genetic markers for reproduction, SNPs in genes that are well known to be involved in reproductive processes, and SNPs in genes reported to be differentially expressed between physiological conditions in a variety of tissues associated in reproductive function. As an additional goal, SNPs were also evaluated for their relationship to other traits. Given the negative genetic correlation between milk yield and reproduction [1-3], it was hypothesized that some SNPs associated with DPR would have an antagonistic relationship with production traits.

## Methods

### Selection of bulls

Straws and ampules of semen were obtained from 550 Holstein bulls born between 1962 and 2010. Bulls were chosen based on their predicted transmitting ability (PTA) and reliability for DPR. In particular, bulls were chosen to have either a high PTA for DPR (≥ 1.7) or low PTA for DPR (≤ −2) with reliability as high as possible. The PTA for the low DPR group (n = 262) ranged from −5.9 to −2 (average = −3.5), and the PTA for the high DPR group (n = 288) ranged from 1.7 to 5.3 (average = 2.87). Reliabilities ranged from 0.46 to 0.99 (3 bulls < 50%, 17 between 50 and 60%, 150 between 60 and 70%, 213 between 70 and 80%, 47 between 80 and 90%, and 120 greater than 90%). The distribution of reliabilities was similar between the low (average = 79%) and high (average = 77%) DPR groups. Predicted transmitting abilities for a variety of traits of the bulls are presented in Additional file 1: Table S1. Semen was obtained from the Cooperative Dairy DNA Repository [CDDR (Beltsville, MD, USA; 445 bulls)], Alta Genetics (Watertown, WI, USA; 38 bulls), Genex Cooperative Inc. (Shawano, WI, USA; 31 bulls), Taurus-service Inc. (Mehoopany, PA, USA; 26 bulls), Foundation Sires Inc. (Listowel, ON, CAN; 5 bulls), Accelerated Genetics (Baraboo, WI, USA, 2 bulls), Interglobe Genetics (Pontiac, IL, USA, 2 bulls), and Nebraska Bull Service (McCook, NE, USA, 1 bull). Five bulls were born in the 1960s, 15 in the 1970s, 54 in the 1980s, 154 in the 1990s, and 322 in the 2000s.

### SNP discovery

The choice of 434 SNPs to be used for genotyping was made using a three-step process: candidate gene selection, SNP identification, and SNP selection. A list of candidate genes affecting reproduction was compiled using two methods. The first was to include genes commonly known to affect reproductive processes such as steroidogenesis (*STAR, HSD17B3*, etc.), follicular development (*LHB, FSHB,* etc.), oocyte maturation (*BMP15, GDF9*, etc.), and early embryonic development (*CSF2, IGF1,* etc.), as well as nutritional genes including orexins (*NPY, HCRT*, etc.) and anorexins (*CCK, LEP*, etc.). Furthermore, genes that were in physical proximity to SNPs related genetically to interval to insemination (*IGFBP7, IRF9,* etc. [28]) and 56 d non-return rate (*BAIAP2, SCRN1,* etc. [29]) were included. In addition, genes reported to be differentially expressed between physiological conditions in a variety of tissues associated with reproductive function were incorporated. This list included genes differentially regulated in the following conditions: the brain of cows displaying strong vs. weak estrus (*CALCR, POMC*, etc. [25]), embryos after cryopreservation (*BAX, DSC2,* etc. [29]), superovulated embryos compared to embryos from unstimulated dams (*GOLGA4, KIT*, etc. [30]), embryos which survived to term compared to embryos that died *in vivo* after embryo transfer (*ATP5A1, OCLN*, etc. [27,31]), embryos treated with CSF2 (*CACNA1G, MADD*, etc. [32]) or IGF1 (*COQ9, CREG1*, etc. [33]) compared to control embryos, embryos cultured *in vitro* in the well-of-the-well system compared to embryos cultured in groups (*CSNK1E, ZP4*, etc. [34]), oocytes compared to 8-cell embryos (*CLIC4, PDGFR*, etc. [35]) and blastocysts (*GJA1, TAF9*, etc. [36]), oocytes at different stages of oocyte maturation (*CPS1F, ZP2*, etc. [37]), endometrium related to embryo survival (*DGKA, BSP3,* etc. [26]), endometrium in lactating cows compared to non-lactating cows (*APBB1, ST13,* etc. [38]) or pregnant cows compared to non-pregnant cows (*ASL, GPLD1,* etc. [38]), cumulus cells regulated by the LH surge (*DHCR24, HAS2,* etc. [39]), at different stages of oocyte maturation (*AP3B1, CLU,* etc. [40]), or from embryos produced *in vivo* embryos compared to embryos produced *in vitro* [*LPL, MAGED1*, etc. [41]), dominant follicles compared to subordinate follicles (*CYP19A1, FST,* etc. [42-45]), liver during the transition period (*ACLY, PCCB,* etc. [46]), mammary tissue during lactation (*ABCA1, INSR,* etc. [47]), and oviduct at diestrus compared to estrus (*C3, OVGP1,* etc. [48]).

Using the procedures described above, a total of 1532 candidate genes were identified. The SNPs in each of these genes were identified by querying the SNP database maintained by the National Center for Biotechnology Information (dbSNP; http://www.ncbi.nlm.nih.gov/snp). Then, SNPs were screened to only include those in the coding region of the gene which resulted in a nonsense, frameshift, or missense mutation. Of the 1532 genes screened, 553 genes containing a total of 1644 SNPs fit those criteria. In addition to these markers, SNPs previously linked to fertility were considered for inclusion. That list of candidate SNPs included *CAST*[15], *FGF2*[16], *FSHR*[19], *GHR*[18], *HSPA1A*[23], *ITGB5*[17], *LEP*[22], *NLRP9*[21], *PAPPA2*[24], *PGR*[14], *SERPINA14*[49], and *STAT5A*[11,16].

In order to determine the final list of SNPs to be used in the assay, each SNP was graded based on primer designability and predicted change in protein function. Each SNP causing an amino acid change was evaluated for the likelihood that the SNP would change the structure of the encoded protein using an exchangeability matrix [50]. The average exchangeability value was calculated for each substitution of pairs of amino acids, and SNPs were ranked in order of exchangeability. For final selection of 434 SNPs, a maximum of one SNP per gene was selected. Nonsense mutations were selected first, then frameshifts, followed by SNPs with the lowest score in the exchangeability matrix (those most likely to cause a change in protein function). The selection criteria were also applied to SNPs already linked genetically to reproduction. Of the final selected SNPs, 5 were the exact SNPs used in the literature: *STAT5A*[11]*, FGF2*[16]*, PGR*[14]*, HSPA1A*[23]*,* and *PAPPA2*[24]*,* and 7 SNPs were replaced with the best option using the criteria mentioned above (*ITGB5, GHR, FSHR, NLRP9, LEP, CAST,* and *SERPINA14*). The final list of genes used in the assay is shown in Additional file 1: Table S2 and the SNPs that were chosen from those genes are shown in Additional file 1: Table S3. The SNP panel included 10 nonsense, 22 frameshift, 397 missense, 1 synonymous, 3 intron region, and 1 promoter region SNPs.

### SNP genotyping

Total DNA was extracted from each straw of semen using the DNeasy Blood and Tissue kit (Qiagen, Valencia, CA, USA) according to the manufacturer’s instructions. Amount of double-stranded DNA was assessed using the Quant-it^TM^ Picogreen® dsDNA kit (Invitrogen, Grand Island, NY, USA), and DNA was resuspended to a concentration of 50 ng/μL. Genotyping was performed by GeneSeek Inc. (Lincoln, NE, USA) using the Sequenom MassARRAY® system (iPLEX GOLD; Sequenom, San Diego, CA, USA) according to the manufacturer’s instructions. The technique is based on the analysis of DNA products using matrix-assisted laser desorption ionization time-of-flight mass spectrometry [51]. The region of DNA containing the SNP was amplified by PCR, a primer extension reaction was performed to generate allele-specific DNA products, and the size and amount of each allele-specific product was determined using chip-based mass spectrometry.

### Quality control

Samples with call rates < 70% were removed from all analyses. The average call rate prior to removing those samples was 88.2%. After removing the failed samples, the average call rate was 91.2%. Reliability was assessed by duplicating 18 SNPs for every DNA sample, and by assaying 63 DNA samples twice. Of the duplicated SNPs, 16 were selected based on interest (*CAST, CSF2, CYP19A1, FGF2, GHR, HSPA1A, IFNT, ITGB5, LEP, LHCGR, NALP9, PAPPA2, PGR, POU5F1, STAT5A,* and *UTMP*) and the other two were selected based on poor primer designability (*ETF1* and *POMC*). The primers for the duplicated SNPs were designed based on the sequence of the opposite DNA strand of where the original primer was designed. The duplicated DNA samples were randomly selected. There was 99.2% identity between SNPs duplicated within an assay and 98.6% identity between duplicated samples. After quality control was assessed, duplicated samples were merged. If any genotype at a given SNP did not match between samples, both genotypes were deleted and treated as a no call. Duplicated SNPs were merged in the same manner. The call rate after merging samples and SNPs was 91.5%.

### Statistical analysis

Minor allele frequency (MAF) was determined using the FREQ procedure of SAS (V9.3; SAS Institute Inc., Cary, NC). Distributions of genotypes were tested for deviation from Hardy-Weinberg equilibrium (HWE) using a chi-square test. In addition, chi-square was used to determine whether MAF differed between high and low DPR bulls.

The association of genetic variants with each trait was evaluated using the MIXED procedure of SAS. The full model included:

$$Y_{i}={\mathrm{byr}}_{j}+{\mathrm{\beta SNP}}_{k}+{\mathrm{POLY}}_{i}+\;\epsilon_{i}$$

where Y_*i*_ is the deregressed PTA of the trait of interest for the *i*^*t*h^ bull (*i* = 1, 2, …, 550) , byr_j_ is the fixed effect of the *j*th birth year (j = 1, 2, …, 5; where birth year is grouped by decade: 1960 to 2010) of the *i*^*t*h^ bull, β is the linear regression coefficient for the k^th^ SNP, SNP_*k*_ is the number of copies (*k* = 0, 1, or 2) of the major allele, POLY_*l*_ is the random polygenic effect (including all available pedigree information) of the *i*^*th*^ bull, and ϵ_*i*_ is the random residual effect. The POLY_*l*_ ~ **A**σ_a_^2^ and ϵ_*i*_ ~ **I**σ_e_^2^, where A is the numerator relationship matrix, I is an identity matrix, σ_a_^2^ is the additive genetic variance of the trait of interest, and σ_e_^2^ is the residual error variance. All of the available pedigree information for each bull was used when modeling the covariance among the polygenic effects [52].

SNP effects were estimated using two analyses. In the first, genotype was considered a continuous variable to determine the allele substitution effect (the linear effect of the number of copies of the major allele; least-squares means represent values for 0,1 and 2 copies of the major allele). In the second, genotype was considered a categorical variable, and an orthogonal contrast was used to estimate dominance effects [(AA + aa)/2 vs. Aa]. SNPs in which the linear or dominance effect was *P* < 0.05 were noted. To control for multiple testing, false discovery rate was controlled for by calculating the Q value using the Q-value package in R [53]. The acceptable false discovery rate for the Q value analysis was chosen as 0.05.

### Pathway analysis

The list of genes significantly related to DPR was subjected to pathway analysis using Ingenuity Pathway Analysis (IPA) software (Ingenuity Systems, http://www.ingenuity.com). The reference set was the Ingenuity Knowledge Base (genes only) and both direct and indirect relationships that were experimentally observed were included. Three analyses were conducted. The first was to identify canonical pathways in which 2 or more genes were overrepresented. The program was also used to build customized networks of genes based on direct and indirect relationships. Finally, upstream regulators in which genes related to DPR were overrepresented were identified. A *P* value of 0.05 or less was considered significant for all analyses.

## Results

### Genetic characteristics of bulls used for genotyping

The range of PTAs for bulls are shown in Additional file 1: Table S1, while the effect of DPR class (high or low) on PTAs are shown in Table 1. Daughter pregnancy rate class had a significant effect on all other traits examined. In particular, bulls in the high DPR class had higher PTAs for heifer conception rate (HCR), cow conception rate (CCR), productive life (PL), net merit (NM), fat percent (FPC), and protein percent (PPC) and lower PTAs for milk yield (MY), fat yield (FY), protein yield (PY), and somatic cell score (SCS) than bulls in the low DPR class (Table 1). Correlations among PTAs are shown in Additional file 2: Table S4. Daughter pregnancy rate was significantly and positively correlated with HCR (0.61), CCR (0.91), PL (0.81), NM (0.49), FPC (0.16), and PPC (0.31) and was significantly and negatively correlated with MY (−0.45), FY (−0.35), PY (−0.34), SCS (−0.55), and birth year (BY; -0.15). These results are consistent with correlations reported earlier [54] for traits included in the lifetime net merit selection index. Since the bulls were selected from the two extremes of DPR, correlations within DPR class (DPRC) were also examined (Additional file 2: Table S5). Within the high DPRC, DPR was positively correlated with HCR and CCR and negatively correlated with NM, MY, FY, PY, and BY. Within the low DPRC, DPR was positively correlated with CCR, PL, and NM and was negatively correlated with SCS and BY.

**Table 1:** Effect of daughter pregnancy rate class (high or low) on predicted transmitting ability for selected traits of bulls used for genotyping

		**Least-squares means**		**Standard error**	
**Trait**	***P*-value**	**High**	**Low**	**High**	**Low**
Daughter pregnancy rate	<.0001	2.86	−3.49	0.04	0.04
Heifer conception rate	<.0001	1.20	−1.00	0.08	0.09
Cow conception rate	<.0001	3.20	−4.16	0.11	0.11
Productive life	<.0001	3.51	−2.96	0.13	0.13
Net merit	<.0001	232.97	−156.40	13.32	13.97
Milk yield	<.0001	−332.21	394.10	42.81	44.90
Fat yield	<.0001	−2.70	16.04	1.60	1.68
Percent fat	0.0008	0.04	0.01	0.01	0.01
Protein yield	<.0001	−3.90	9.90	1.18	1.23
Percent protein	<.0001	0.02	−0.01	0.00	0.00
Somatic cell score	<.0001	2.83	3.07	0.01	0.01

### Minor allele frequencies

Of the 434 SNPs, only 107 had MAF ≥ 5% and only 98 of those that had MAF ≥ 5% and had a call rate > 70%. Nine SNPs had MAF ≥ 5% but failed the genotyping process (call rate < 70%; *AHCYL2, APBB1IP, FXC1, HSF1, PHGDH, POMC, SLC1A5, ST13*, and *TTF1*) and were removed from all further analyses. The probability that the MAF was ≥ 5% was dependent upon the type of SNP. Four of the 5 genes in which the SNP was in the non-coding regions or was synonymous had a MAF ≥ 5% (80%) whereas only 20% (2/10) of the nonsense, 25% of the missense (99/397), and 9% (2/22) of the frameshift mutations had ≥ 5% MAF (χ^2^ for non-coding/synonymous vs others, 8.34, *P* < 0.01).

### Hardy-Weinberg equilibrium

Characteristics of the 98 SNPs in which MAF ≥ 5% and call rate was > 70% are shown in Additional file 2: Table S6. A total of 26 SNPs were not in equilibrium (*AVP, BOLA-DMB, C17H22orf25, CCDC88B, CCT8, CD2, CFDP2, COQ9, DEPDC7, DTX2, FUT1, HSD17B6, IBSP, IFNT2, MARVELD1, NEU3, RALGPS1, SEC14L1, SREBF1, STAT5A, SYTL2, TAF9, TSPYL1, UHRF1, WBP1,* and *ZP2*). All but one of these SNPs caused a missense mutation. The exception was for *UHRF1*, which was a frameshift mutation where the mutation causing the frameshift had a frequency of 91.7%. The genes most out of equilibrium were *CCT8, MARVELD1* and *SYTL2*, in which the number of minor allele homozygotes was lower than expected, *CD2, DTX2, NEU3*, and *RALGPS1*, in which the number of heterozygotes was lower than expected, and *TAF9* and *TSPYL1*, in which the number of heterozygotes was greater than expected.

### SNP effects on daughter pregnancy rate

Each of the 98 SNPs with MAF ≥ 5% and a call rate > 70% were analyzed for effects on DPR and other genetic traits. Two types of analyses were performed: a regression analysis to determine the allele substitution effect of each SNP (0, 1 or 2 copies of the major allele) and use of an orthogonal contrast to determine the dominance effect (heterozygote vs. the average of the two homozygotes). Both *P* values and Q values corrected for multiple testing were determined. Since the Q value correction for multiple testing is highly conservative in cases where few tests are significant, both the *P* value and the Q value were used to identify SNPs associated with genetic traits.

Results for DPR are shown in Table 2. Allele substitution effects were different from 0 for 40 genes [*ACAT2, AP3B1, APBB1, BSP3, C17H22orf25* (interim symbol *TANGO2), C1QB, C7H19orf60, CACNA1D, CAST, CCDC86, CD14, CD40, CFDP2, COQ9, CPSF1, CSNK1E, CSPP1, DEPDC7, DSC2, DYRK3, FUT1, GPLD1, HSD17B12, HSD17B7, LDB3, MARVELD1, MON1B, MRGPRF, MS4A8B, NEU3, NFKBIL1, NLRP9, OCLN, PARM1, PCCB, PMM2, RABEP2, TBC1D24, TDRKH,* and *ZP2*]. These effects were significant based on both *P* and Q values. In addition, there were 4 genes exhibiting dominance based on *P* values, including two in which the allele substitution effect was significant (*CD14* and *FUT1*) and two in which the allele substitution was not significant (*ARL6IP1* and *TSHB*). After correcting for multiple testing, none of the dominance effects achieved significance.

**Table 2:** **SNPs associated with daughter pregnancy rate**^**a**^

**SNP**	**Gene**	**Least-squares means (SEM)**			**Linear**			**Dominance**	
		**0**	**1**	**2**	**Effect**	***P *value**	**Q value**	***P *value**	**Q value**
rs109967779	*ACAT2*	0.85 (.58)	0.33 (0.36)	−0.98 (0.37)	−1.00	0.0015	0.0040	0.4072	0.5155
rs133700190	*AP3B1*	1.67 (1.04)	0.46 (0.42)	−0.70 (0.32)	−1.17	0.0026	0.0058	0.9680	0.5492
rs41766835	*APBB1*	0.20 (1.05)	0.78 (0.44)	−0.63 (0.31)	−0.95	0.0163	0.0175	0.1417	0.5155
rs110541595	*ARL6IP1*	−0.40 (0.58)	0.63 (0.37)	−0.92 (0.41)	−0.50	0.1337	0.0849	0.0079	0.4213
rs110217852	*BSP3*	−1.82 (0.73)	−0.21 (0.37)	0.24 (0.35)	0.81	0.0169	0.0176	0.2764	0.5155
rs133455683	*C17H22orf25*	0.83 (0.53)	−0.16 (0.37)	−0.76 (0.40)	−0.77	0.0150	0.0166	0.6773	0.5155
rs135390325	*C1QB*	−3.43 (3.62)	−1.23 (0.53)	0.25 (0.28)	1.51	0.0061	0.0091	0.8484	0.5155
rs109332658	*C7H19orf60*	−1.40 (0.84)	−0.37 (0.39)	0.20 (0.34)	0.69	0.0558	0.0478	0.6871	0.5155
rs135744058	*CACNA1D*	0.29(0.88)	0.69 (0.37)	−0.81 (0.32)	−1.03	0.0038	0.0072	0.1000	0.5155
rs137601357	*CAST*	−1.35 (0.52)	−0.45 (0.35)	0.79 (0.41)	1.09	0.0004	0.0015	0.6970	0.5155
rs109447102	*CCDC86*	−1.48 (1.10)	−0.74 (0.44)	0.33 (0.31)	1.00	0.0125	0.0153	0.8179	0.5155
rs109621328	*CD14*	6.90 (2.10)	0.65 (0.63)	−0.28 (0.28)	−1.66	0.0043	0.0072	0.0291	0.5155
rs41711496	*CD40*	−0.76 (0.45)	0.03 (0.35)	0.73 (0.45)	0.74	0.0150	0.0166	0.9815	0.5510
rs41857027	*CFDP2*	−2.53 (0.80)	0.04 (0.57)	0.34 (0.30)	0.92	0.0029	0.0058	0.1016	0.5155
rs109301586	*COQ9*	−1.42 (0.44)	−0.16 (0.39)	1.16 (0.42)	1.29	<0.0001	0.0006	0.9458	0.5426
rs134432442	*CPSF1*	−1.90 (0.94)	−0.36 (0.41)	0.24 (0.33)	0.82	0.0255	0.0239	0.4347	0.5155
rs133449166	*CSNK1E*	0.60 (0.55)	−0.22 (0.36)	−0.86 (0.40)	−0.72	0.0224	0.0226	0.8492	0.5155
rs109443582	*CSPP1*	−0.61 (3.63)	−1.68 (0.65)	−1.68 (0.29)	1.61	0.0131	0.0155	0.4607	0.5155
rs110270752	*DEPDC7*	−2.66 (0.94)	−0.69 (0.43)	0.31 (0.31)	1.25	0.0010	0.0031	0.4458	0.5155
rs109503725	*DSC2*	−1.06 (0.45)	−0.14 (0.35)	0.52 (0.45)	0.79	0.0099	0.0136	0.7673	0.5155
rs109561866	*DYRK3*	−1.58 (2.06)	−1.06 (0.51)	−0.06 (0.29)	0.95	0.0538	0.0473	0.8323	0.5155
rs41893756	*FUT1*	−1.08 (0.95)	−1.51 (0.44)	0.61 (0.30)	1.47	0.0001	0.0006	0.0448	0.5155
rs109516714	*GPLD1*	−1.60 (0.57)	−0.22 (0.36)	0.38 (0.40)	0.92	0.0043	0.0072	0.4062	0.5155
rs109711583	*HSD17B12*	0.76 (0.52)	−0.04 (0.34)	−0.69 (0.42)	−0.72	0.0258	0.0239	0.08603	0.5155
rs110828053	*HSD17B7*	0.79 (1.09)	0.80 (0.42)	−0.62 (0.31)	−1.12	0.0044	0.0072	0.2996	0.5155
rs111015912	*LDB3*	2.43 (1.03)	0.80 (0.39)	−0.74 (0.31)	−1.51	<0.0001	0.0006	0.9462	0.5426
rs134011564	*MARVELD1*	−0.33 (3.63)	0.07 (0.32)	−1.76 (0.67)	−1.75	0.0107	0.0141	0.5477	0.5155
rs41859871	*MON1B*	4.57 (2.06)	0.78 (0.49)	−0.47 (0.29)	−1.50	0.0019	0.0047	0.2680	0.5155
rs109248655	*MRGPRF*	N/A N/A	−1.42 (0.63)	0.03 (0.28)	1.45	0.0288	0.0260	N/A	N/A
rs109761676	*MS4A8B*	−1.78 (0.88)	−0.77 (0.37)	0.65 (0.34)	1.31	0.0004	0.0015	0.7240	0.5155
rs133762601	*NEU3*	−1.51 (0.60)	−1.39 (0.94)	0.07 (0.30)	0.84	0.0064	0.0091	0.4928	0.5155
rs133497176	*NFKBIL1*	−1.22 (1.38)	−1.31 (0.50)	0.11 (0.28)	1.14	0.0117	0.0149	0.3687	0.5155
rs109383758	*NLRP9*	0.57 (0.45)	−0.22 (0.34)	−0.78 (0.45)	−0.67	0.0253	0.0239	0.7887	0.5155
rs134264563	*OCLN*	0.92 (0.80)	0.31 (0.36)	−0.81 (0.34)	−0.98	0.0048	0.0075	0.6312	0.5155
rs111027720	*PARM1*	2.21 (0.47)	0.11 (0.34)	−2.38 (0.40)	−2.31	<0.0001	0.0006	0.6458	0.5155
rs109813896	*PCCB*	1.51 (0.62)	0.11 (0.36)	−0.71 (0.37)	−1.02	0.0014	0.0040	0.5355	0.5155
rs109629628	*PMM2*	1.43 (0.61)	0.03 (0.35)	−0.99 (0.38)	−1.16	0.0004	0.0015	0.6895	0.5155
rs133729105	*RABEP2*	−1.36 (0.57)	−0.38 (0.36)	0.53 (0.37)	0.94	0.0027	0.0058	0.9448	0.5426
rs110660625	*TBC1D24*	1.21 (0.65)	0.32 (0.37)	−0.89 (0.36)	−1.10	0.0006	0.0021	0.7474	0.5155
rs110805802	*TDRKH*	−6.62 (1.59)	−1.64 (0.49)	0.67 (0.29)	2.70	<0.0001	0.0006	0.1513	0.5155
rs132789482	*TSHB*	−3.05 (1.49)	0.50 (0.56)	0.40 (0.33)	0.60	0.2270	0.1145	0.0493	0.5155
rs110883602	*ZP2*	−1.83 (0.56)	−0.11 (0.42)	1.09 (0.40)	1.42	<0.0001	0.0006	0.6224	0.5155

^a^Single nucleotide polymorphism represented as the rs number given by the National Center for Biotechnology Information data base SNP.

### SNP effects on other fertility traits

For HCR, there were allele substitution effects for 19 SNPs (*AP3B1, APBB1, C1QB, CACNA1D, CD14, CPSF1, CSNK1E, DEPDC7, DSC2, FSHR, FYB, GPLD1, HSD17B7, LDB3, MS4A8B, NFKBIL1, PARM1, TDRKH,* and *ZP2*) and dominance effects for 5 SNPs (*ARPL6IP1,CACNA1D, CD14, DZIP3,* and *GOLGA4*; Table 3). None of the dominant effects remained significant after correcting for multiple testing. The only SNPs significant after correcting for multiple testing were allele substitution effects for *DEPDC7, LDB3, MS4A8B, PARM1,* and *TDRKH*.

**Table 3:** **SNPs associated with heifer conception rate**^**a**^

**SNP**	**Gene**	**Least-squares means (SEM)**			**Linear**			**Dominance**	
		**0**	**1**	**2**	**Effect**	***P *value**	**Q value**	***P *value**	**Q value**
rs133700190	*AP3B1*	3.74 (1.59)	2.72 (0.63)	1.24 (0.46)	−1.38	0.0204	0.1290	0.8236	0.9592
rs41766835	*APBB1*	2.37 (1.40)	2.49 (0.61)	1.13 (0.44)	−1.02	0.0288	0.1410	0.4152	0.9592
rs110541595	*ARL6IP1*	1.28 (0.87)	3.36 (0.53)	0.50 (0.59)	−0.85	0.0977	0.2380	0.0010	0.0713
rs135390325	*C1QB*	2.37 (1.40)	2.49 (0.61)	1.13 (0.44)	1.87	0.0224	0.1290	0.7779	0.9592
rs135744058	*CACNA1D*	2.00 (1.30)	3.42 (0.56)	0.95 (0.49)	−1.51	0.0041	0.0519	0.0229	0.5439
rs109621328	*CD14*	12.97 (3.16)	2.34 (0.95)	1.65 (0.41)	−2.05	0.0192	0.1290	0.0068	0.2153
rs134432442	*CPSF1*	−0.61 (1.44)	1.18 (0.61)	2.35 (0.48)	1.31	0.0212	0.1290	0.7404	0.9592
rs133449166	*CSNK1E*	2.82 (0.82)	2.15 (0.53)	0.86 (0.59)	−1.03	0.0321	0.1410	0.6649	0.9592
rs110270752	*DEPDC7*	−1.71 (1.44)	0.64 (0.65)	2.66 (0.47)	2.10	0.0003	0.0095	0.8625	0.9592
rs109503725	*DSC2*	0.88 (0.67)	1.76 (0.51)	2.79 (0.66)	0.95	0.0386	0.1438	0.9054	0.9592
rs133175991	*DZIP3*	6.52 (1.77)	1.76 (0.63)	1.44 (0.45)	−1.18	0.0556	0.1677	0.0435	0.6897
rs43745234	*FSHR*	−0.38 (1.14)	1.52 (0.53)	2.53 (0.51)	1.27	0.0149	0.1290	0.5717	0.9592
rs109262355	*FYB*	1.17 (0.87)	0.94 (0.53)	2.77 (0.55)	1.04	0.0302	0.1410	01484	0.7832
rs42339105	*GOLGA4*	−17.26 (5.65)	1.73 (0.87)	1.78 (0.40)	0.95	0.3028	0.3760	0.0015	0.0713
rs109516714	*GPLD1*	0.42 (0.85)	1.63 (0.53)	2.52 (0.59)	1.02	0.0369	0.1438	0.8243	0.9592
rs110828053	*HSD17B7*	1.74 (1.64)	3.15 (0.62)	1.17 (0.44)	−1.27	0.0334	0.1410	0.1038	0.6897
rs111015912	*LDB3*	4.41 (1.57)	2.72 (0.59)	1.16 (0.46)	−1.59	0.0067	0.0707	0.9491	0.9592
rs109761676	*MS4A8B*	−0.58 (1.29)	0.94 (0.55)	2.95 (0.50)	1.88	0.0006	0.0127	0.7764	0.9592
rs133497176	*NFKBIL1*	−1.09 (2.07)	0.82 (0.74)	2.04 (0.42)	1.35	0.0474	0.1580	0.7885	0.9592
rs111027720	*PARM1*	3.78 (0.72)	2.19 (0.52)	−0.28 (0.62)	−2.06	<0.0001	0.0057	0.5129	0.9592
rs110805802	*TDRKH*	−6.06 (2.48)	0.90 (0.77)	2.53 (0.44)	2.42	0.0010	0.0158	0.0673	0.6897
rs110883602	*ZP2*	0.48 (0.81)	1.76 (0.61)	2.45 (0.59)	0.94	0.0433	0.1524	0.6904	0.9592

^a^Single nucleotide polymorphism represented as the rs number given by the National Center for Biotechnology Information database SNP.

For CCR, there were allele substitution effects for 29 SNPs (*ACAT2, AP3B1, APBB1, BCAS1, C1QB, CAST, CCDC86, CCT8, CFDP2, COQ9, CPSF1, CSNK1E, CSPP1, FUT1, GPLD1, HSD17B7, LDB3, MARVELD1, MON1B, NEU3, NFKBIL1, OCLN, PARM1, PMM2, RABEP2, TBC1D24, TDRKH, WBP1,* and *ZP2*) and dominance effects for 4 SNPs (*ARL6IP1, SEC14L1, SERPINE2,* and *SLC18A2*; Table 4). All but one of the allele substitution effects were significant after correction for multiple testing, the exception being for *ARL6IP1*, but none of the dominance effects were significant based on Q values.

**Table 4:** **SNPs associated with cow conception rate**^**a**^

**SNP**	**Gene**	**Least-squares means (SEM)**			**Linear**			**Dominance**	
		**0**	**1**	**2**	**Effect**	***P *value**	**Q value**	***P *value**	**Q value**
rs109967779	*ACAT2*	1.89 (1.09)	1.03 (1.03)	−0.61 (−0.61)	−1.34	0.0216	0.0334	0.6559	0.3915
rs133700190	*AP3B1*	3.89 (1.95)	1.93 (0.78)	−0.82 (0.59)	−2.58	0.0004	0.0023	0.7487	0.3915
rs41766835	*APBB1*	2.78 (1.98)	1.64 (0.84)	−0.19 (0.60)	−1.67	0.0255	0.0361	0.7889	0.3915
rs110541595	*ARL6IP1*	−0.23 (1.09)	1.90 (0.69)	−0.97 (0.76)	−0.84	0.1781	0.1081	0.0059	0.1180
rs109669573	*BCAS1*	−1.68 (1.00)	1.01 (0.67)	1.16 (0.76)	1.24	0.0359	0.0421	0.1387	0.3915
rs135390325	*C1QB*	−2.74 (6.74)	−2.20 (0.99)	1.26 (0.51)	3.35	0.0012	0.0058	0.6761	0.3915
rs137601357	*CAST*	−1.33 (0.98)	0.06 (0.65)	2.01 (0.76)	1.71	0.0032	0.0086	0.7283	0.3915
rs109447102	*CCDC86*	−2.22 (2.06)	−0.40 (0.82)	1.14 (0.57)	1.60	0.0324	0.0408	0.9181	0.3992
rs137673698	*CCT8*	−10.00 (9.38)	0.18 (0.52)	4.14 (1.66)	4.16	0.0120	0.0204	0.5150	0.3915
rs41857027	*CFDP2*	−3.70 (1.31)	0.78 (0.93)	0.87 (0.48)	1.65	0.0068	0.0128	0.0517	0.3915
rs109301586	*COQ9*	−1.53 (0.83)	0.51 (0.73)	2.58 (0.79)	2.06	0.0002	0.0014	0.9796	0.4125
rs134432442	*CPSF1*	−3.08 (1.77)	−0.14 (0.76)	1.26 (0.60)	1.77	0.0111	0.0199	0.5051	0.3915
rs133449166	*CSNK1E*	2.02 (1.03)	0.77 (0.67)	−1.42 (0.75)	−1.80	0.0026	0.0086	0.5917	0.3915
rs109443582	*CSPP1*	1.04 (6.88)	−2.44 (1.23)	0.97 (0.56)	3.01	0.0026	0.0086	0.5917	0.3915
rs41893756	*FUT1*	−1.16 (1.79)	−1.29 (0.82)	1.56 (0.57)	2.10	0.0033	0.0086	0.2157	0.3915
rs109516714	*GPLD1*	−2.36 (1.06)	0.67 (0.68)	1.40 (0.75)	1.67	0.0055	0.0110	0.1921	0.3915
rs110828053	*HSD17B7*	1.96 (2.01)	2.17 (0.78)	−0.55 (0.57)	−2.10	0.0039	0.0091	0.2472	0.3915
rs111015912	*LDB3*	4.74 (1.94)	1.56 (0.74)	−0.28 (0.59)	−2.12	0.0029	0.0086	0.5836	0.3915
rs134011564	*MARVELD1*	2.54 (6.20)	0.72 (0.54)	−2.83 (1.13)	−3.48	0.0029	0.0086	0.7852	0.3915
rs41859871	*MON1B*	7.15 (3.85)	1.56 (0.92)	−0.01 (0.54)	−1.96	0.0296	0.0387	0.3455	0.3915
rs133762601	*NEU3*	−2.06 (1.13)	−1.56 (1.76)	1.03 (0.56)	1.62	0.0050	0.0106	0.5700	0.3915
rs133497176	*NFKBIL1*	−2.45 (2.57)	−1.24 (0.93)	0.81 (0.54)	1.90	0.0251	0.0361	0.7873	0.3915
rs134264563	*OCLN*	1.70 (1.49)	1.11 (0.68)	−0.57 (0.63)	−1.37	0.0352	0.0421	0.5920	0.3915
rs111027720	*PARM1*	4.31 (0.89)	0.39 (0.64)	−2.30 (0.77)	−3.25	<0.0001	0.0010	0.4499	0.3915
rs109629628	*PMM2*	3.09 (1.13)	1.10 (0.64)	−1.29 (0.71)	−2.24	0.0002	0.0014	0.8245	0.3915
rs133729105	*RABEP2*	−1.44 (1.07)	−0.14 (0.67)	1.79 (0.70)	1.68	0.004	0.0091	0.7211	0.3915
rs136746215	*SEC14L1*	−1.70 (1.02)	3.44 (1.23)	0.83 (0.72)	0.98	0.0988	0.0819	0.0037	0.1180
rs43321188	*SERPINE2*	4.29 (1.51)	0.09 (0.73)	0.24 (0.60)	−1.10	0.0972	0.0819	0.0389	0.3890
rs110365063	*SLC18A2*	7.68 (2.61)	0.44 (0.86)	0.41 (0.57)	−1.15	0.1468	0.0998	0.0197	0.2627
rs110660625	*TBC1D24*	2.36 (1.21)	1.49 (0.69)	−0.90 (0.67)	−1.86	0.0021	0.0086	0.4047	0.3915
rs110805802	*TDRKH*	−10.71 (3.00)	−2.18 (0.92)	1.66 (0.53)	4.53	<0.0001	0.0010	0.1826	0.3915
rs134282928	*WBP1*	3.78 (5.57)	−1.21 (0.89)	1.16 (0.58)	2.03	0.0296	0.0387	0.2062	0.3915
rs110883602	*ZP2*	−2.77 (1.10)	0.94 (0.81)	2.76 (0.78)	2.62	<0.0001	0.0010	0.3468	0.3915

^a^Single nucleotide polymorphism represented as the rs number given by the National Center for Biotechnology Information database SNP.

### SNP effects on productive life and net merit

For PL, there were allele substitution effects for 33 SNPs (*ACAT2, AP3B1, ASL, CCDC86, CD40, CFDP2, COQ9, CSPP1, DEPDC7, DSC2, FSHR, FUT1, GPLD1, HSD17B12, HSD17B6, HSD17B7, HSPA1A, LDB3, LHCGR, MARVELD1, MON1B, MS4A8B, NEU3, OCLN, PARM1, PCCB, PMM2, RABEP2, SYTL2, TBC1D24, TDRKH, WBP1,* and *ZP2*) and dominance effects for 5 SNPs (*ARL6IP1, AVP, CSPP1, DEPDC7,* and *IBSP*; Table 5). After correcting for multiple testing, none of the dominant effects were significant.

**Table 5:** **SNPs associated with productive life**^**a**^

**SNP**	**Gene**	**Least-squares means (SEM)**			**Linear**			**Dominance**	
		**0**	**1**	**2**	**Effect**	***P *value**	**Q value**	***P *value**	**Q value**
rs109967779	*ACAT2*	2.05 (0.65)	1.24 (0.41)	−0.18 (0.42)	−1.18	0.0006	0.0014	0.5495	0.9408
rs133700190	*AP3B1*	3.74 (1.11)	1.16 (0.47)	0.15 (0.36)	−1.42	0.0006	0.0014	0.2684	0.9053
rs110541595	*ARL6IP1*	0.22 (0.63)	1.49 (0.41)	−0.12 (0.45)	−0.45	0.2040	0.1193	0.0051	0.4845
rs110127056	*ASL*	1.49 (0.55)	0.90 (0.40)	−0.11 (0.47)	−0.82	0.0159	0.0204	0.6685	0.9798
rs43114141	*AVP*	0.23 (0.56)	1.24 (0.45)	0.15 (0.47)	−0.12	0.7144	0.2797	0.0446	0.8474
rs109447102	*CCDC86*	−0.42 (1.21)	−0.16 (0.49)	1.17 (0.35)	1.18	0.0074	0.0112	0.6685	0.9798
rs41711496	*CD40*	−0.50 (0.50)	0.71 (0.40)	2.28 (0.50)	1.39	<0.0001	0.0004	0.4864	0.9408
rs41857027	*CFDP2*	−0.69 (0.90)	0.87 (0.64)	1.27 (0.36)	0.81	0.0482	0.0489	0.4461	0.9408
rs109301586	*COQ9*	−0.67 (0.50)	0.71 (0.44)	2.34 (0.48)	1.50	<0.0001	0.0004	0.8009	0.9798
rs109443582	*CSPP1*	3.16 (3.99)	−1.02 (0.72)	1.08 (0.34)	1.74	0.0147	0.0204	0.0124	0.5059
rs110270752	*DEPDC7*	−3.30 (1.02)	0.48 (0.48)	1.09 (0.36)	1.41	0.0005	0.0014	0.0202	0.5059
rs109503725	*DSC2*	0.28 (0.51)	0.35 (0.40)	1.61 (0.50)	0.67	0.0445	0.0478	0.2216	0.9053
rs43745234	*FSHR*	−1.14 (0.82)	0.36 (0.40)	1.34 (0.39)	1.13	0.0023	0.0045	0.6438	0.9708
rs41893756	*FUT1*	−0.86 (1.04)	−0.96 (0.49)	1.60 (0.35)	1.88	<0.0001	0.0004	0.0546	0.8487
rs109516714	*GPLD1*	−0.34 (0.63)	0.55 (0.41)	1.17 (0.45)	0.73	0.0351	0.0403	0.7998	0.9798
rs109711583	*HSD17B12*	2.01 (0.57)	0.80 (0.39)	−0.08 (0.47)	−1.03	0.0030	0.0053	0.7318	0.9798
rs109769865	*HSD17B6*	0.25 (1.42)	−0.80 (0.56)	1.16 (0.34)	1.35	0.0054	0.0090	0.0915	0.8487
rs110828053	*HSD17B7*	2.01 (1.18)	1.24 (0.47)	0.34 (0.35)	−0.87	0.0397	0.0441	0.9303	0.9830
HSP70C895D	*HSPA1A*	1.39 (0.84)	1.27 (0.43)	0.07 (0.38)	−0.90	0.0155	0.0204	0.3526	0.9053
rs110789098	*IBSP*	0.65 (0.56)	1.36 (0.42)	−0.25 (0.47)	−0.58	0.0888	0.0664	0.0213	0.5059
rs111015912	*LDB3*	3.01 (1.10)	1.87 (0.44)	−0.12 (0.36)	−1.81	<0.0001	0.0004	0.5322	0.9408
rs41256848	*LHCGR*	1.97 (0.60)	0.71 (0.38)	0.03 (0.46)	−0.94	0.0061	0.0097	0.5546	0.9408
rs134011564	*MARVELD1*	2.34 (3.77)	0.81 (0.36)	−0.87 (0.71)	−1.67	0.0188	0.0232	0.9690	0.9830
rs41859871	*MON1B*	5.54 (2.25)	1.72 (0.55)	0.31 (0.33)	−1.65	0.0018	0.0040	0.3340	0.9053
rs109761676	*MS4A8B*	−1.96 (0.96)	0.33 (0.43)	1.46 (0.39)	1.43	0.0004	0.0014	0.3471	0.9053
rs133762601	*NEU3*	−0.81 (0.66)	−1.29 (1.02)	0.98 (0.34)	0.99	0.0030	0.0053	0.1963	0.9053
rs134264563	*OCLN*	1.84 (0.88)	1.03 (0.41)	0.01 (0.38)	−0.96	0.0113	0.0164	0.8560	0.9798
rs111027720	*PARM1*	3.06 (0.54)	0.77 (0.39)	−1.03 (0.46)	−2.03	<0.0001	0.0004	0.6161	0.9708
rs109813896	*PCCB*	2.00 (0.68)	1.05 (0.41)	0.07 (0.41)	−1.06	0.0023	0.0045	0.9700	0.9830
rs109629628	*PMM2*	3.26 (0.66)	0.94 (0.39)	−0.48 (0.43)	−1.76	<0.0001	0.0004	0.3833	0.9408
rs133729105	*RABEP2*	−0.86 (0.62)	0.25 (0.40)	1.68 (0.42)	1.30	<0.0001	0.0004	0.7495	0.9798
rs42158454	*SYTL2*	N/A N/A	1.88 (0.63)	0.42 (0.33)	−1.46	0.0261	0.0311	N/A	N/A
rs110660625	*TBC1D24*	2.12 (0.70)	1.00 (0.41)	−0.11 (0.39)	−1.17	0.0006	0.0014	0.9830	0.9830
rs110805802	*TDRKH*	−4.35 (1.72)	−0.47 (0.55)	1.20 (0.33)	2.00	<0.0001	0.0004	0.2737	0.9053
rs134282928	*WBP1*	2.73 (3.26)	−0.26 (0.53)	1.01 (0.35)	1.07	0.0484	0.0489	0.2112	0.9053
rs110883602	*ZP2*	−1.06 (0.64)	1.05 (0.48)	1.67 (0.47)	1.25	0.0005	0.0014	0.1876	0.9053

^a^Single nucleotide polymorphism represented as the rs number given by the National Center for Biotechnology Information database SNP. For entries not beginning with rs, the abbreviation given by previous researchers was used.

For NM, there were allele substitution effects for 30 SNPs (*ACAT2, AP3B1, ASL, C17H22orf25, CCT8, CD2, CD40, COQ9, CSNK1E, DEPDC7, EPAS1, FST, FUT1, HSD17B12, HSD17B6, HSPA1A, IBSP, LDB3, LHCGR, MON1B, MRPL48, MS4A8B, NEU3, OCLN, PARM1, PCCB, PMM2, RABEP2, TBC1D24,* and *TDRKH*) and dominance effects for 6 SNPs (SNPs in *ARL6IP1, CD14, DEPDC7, FGF2, IBSP,* and *SLC18A2*; Table 6). Except for *HSPA1A*, the allele substitution effects were significant after correcting for multiple testing, but dominance effects were not significant.

**Table 6:** **SNPs associated with net merit**^**a**^

**SNP**	**Gene**	**Least-squares means (SEM)**			**Linear**			**Dominance**	
		**0**	**1**	**2**	**Effect**	***P *value**	**Q value**	***P *value**	**Q value**
rs109967779	*ACAT2*	178.42 (48.24)	98.35 (30.35)	15.01 (31.97)	−82.07	0.0012	0.0070	0.9660	0.8362
rs133700190	*AP3B1*	263.47 (82.15)	105.28 (35.29)	39.78 (27.83)	−86.36	0.0043	0.0134	0.3700	0.7538
rs110541595	*ARL6IP1*	19.00 (47.24)	102.08 (31.27)	34.27 (33.92)	−7.02	0.7872	0.4223	0.0471	0.6280
rs110127056	*ASL*	126.52 (40.99)	92.90 (30.18)	15.06 (35.27)	−58.04	0.0190	0.0386	0.5267	0.7538
rs133455683	*C17H22orf25*	153.24 (44.49)	81.81 (32.19)	33.74 (34.16)	−58.15	0.0227	0.0441	0.7567	0.8181
rs137673698	*CCT8*	−66.96 (384.43)	64.02 (25.15)	218.87 (70.34)	154.21	0.0254	0.0456	0.9513	0.8362
rs109621328	*CD14*	−330.45 (164.59)	75.08 (51.21)	92.99 (24.82)	71.41	0.1123	0.1167	0.0429	0.6280
rs133747802	*CD2*	25.05 (37.20)	51.73 (57.91)	124.90 (31.41)	50.51	0.0178	0.0378	0.6950	0.7831
rs1711496	*CD40*	−34.02 (37.54)	78.45 (30.39)	177.35 (37.44)	105.65	<0.0001	0.0011	0.8510	0.8362
rs109301586	*COQ9*	12.99 (36.38)	76.86 (32.52)	180.47 (35.29)	83.46	0.0002	0.0016	0.5816	0.7538
rs133449166	*CSNK1E*	148.36 (45.78)	74.03 (31.33)	5.10 (34.49)	−71.12	0.0057	0.0166	0.9420	0.8362
rs110270752	*DEPDC7*	−205.46 (76.20)	57.40 (35.94)	89.30 (27.20)	90.54	0.0028	0.0104	0.0236	0.6280
rs43676052	*EPAS1*	47.14 (94.81)	10.96 (33.14)	121.94 (27.62)	84.96	0.0077	0.0211	0.1892	0.7538
FGF2ag	*FGF2*	31.03 (49.04)	118.22 (31.03)	43.39 (31.60)	−12.88	0.6165	0.3509	0.0341	0.6280
rs109247499	*FST*	142.59 (37.96)	75.63 (30.09)	−3.82 (37.54)	−73.25	0.0023	0.0102	0.8578	0.8362
rs41893756	*FUT1*	−54.03 (75.53)	−39.78 (36.51)	134.76 (26.92)	133.31	<0.0001	0.0011	0.1074	0.7538
rs109711583	*HSD17B12*	168.57 (42.46)	73.40 (29.38)	18.20 (35.19)	−72.89	0.0041	0.0134	0.5686	0.7538
rs109769865	*HSD17B6*	−17.39 (104.02)	−12.91 (41.89)	(25.95)	92.53	0.0091	0.0222	0.3951	0.7538
HSP70C895D	*HSPA1A*	102.78 (62.61)	107.06 (32.41)	27.50 (28.95)	−55.77	0.0417	0.0671	0.3526	0.9053
rs110789098	*IBSP*	75.84 (42.40)	127.06 (32.00)	−15.18 (35.38)	−56.10	0.0276	0.0460	0.0105	0.5680
rs111015912	*LDB3*	180.77 (83.38)	129.65 (33.53)	23.71 (27.55)	−94.51	0.0018	0.0093	0.5936	0.7538
rs41256848	*LHCGR*	144.34 (44.56)	66.31 (29.33)	37.07 (34.33)	−49.65	0.0452	0.0703	0.5546	0.9408
rs41859871	*MON1B*	459.24 (163.85)	148.99 (40.47)	43.67 (25.54)	−125.76	0.0010	0.0067	0.2594	0.7538
rs43703916	*MRPL48*	13.22 (41.27)	88.01 (30.70)	118.26 (40.09)	52.03	0.0488	0.0735	0.461	0.9408
rs109761676	*MS4A8B*	−72.05 (71.38)	50.4 (33.00)	112.45 (30.27)	77.88	0.0095	0.0222	0.5108	0.7538
rs133762601	*NEU3*	−23.09 (49.33)	−94.09 (75.68)	83.89 (26.51)	61.77	0.0115	0.0256	0.1105	0.7538
rs134264563	*OCLN*	147.95 (65.06)	84.23 (31.17)	21.84 (29.23)	−62.76	0.024	0.0448	0.9878	0.8362
rs111027720	*PARM1*	209.58 (41.04)	73.79 (30.13)	−18.2 (35.56)	−111.8	<0.0001	0.0011	0.5531	0.7538
rs109813896	*PCCB*	177.33 (50.88)	99.87 (30.79)	21.02 (30.85)	−78.35	0.0024	0.0102	0.9856	0.8362
rs109629628	*PMM2*	290.06 (49.07)	103.79 (29.91)	−34.46 (32.43)	−156.4	<0.0001	0.0011	0.5195	0.7538
rs133729105	*RABEP2*	−8.85 (46.79)	48.97 (30.72)	117.46 (31.97)	64.29	0.0088	0.0222	0.8863	0.8362
rs110365063	*SLC18A2*	−63.33 (115.05)	160.24 (38.53)	50.69 (26.17)	−57.22	0.0998	0.1136	0.0142	0.568
rs110660625	*TBC1D24*	178.3 (52.52)	71.03 (31.02)	12.25 (29.81)	−75.7	0.0029	0.0104	0.528	0.7538
rs110805802	*TDRKH*	−280.61 (126.98)	−25.67 (41.26)	95.88 (25.52)	141.8	0.0002	0.0016	0.3684	0.7538

^a^Single nucleotide polymorphism represented as the rs number given by the National Center for Biotechnology Information database SNP. For entries not beginning with rs, the abbreviation given by previous researchers was used.

### SNP effects on production traits

There were fewer effects on production traits compared to fertility traits, which is consistent with the conclusion of Cole et al. [55] that yield traits generally are consistent with an infinitesimal model, in which the trait is controlled by many alleles of small effect. For MY, there were allele substitution effects for 18 SNPs and dominance effects for 6 SNPs (Table 7). Only linear effects of *CD14, CPSF1, FAM5C,* and *PARM1* were significant after correcting for multiple testing. For FY, there were allele substitution effects for 13 SNPs and dominance effects for 7 SNPs (Table 8). Only the linear effects of *CPSF1* and *PARM1* were significant after correcting for multiple testing. For FPC, there were allele substitution effects for 10 SNPs and dominance effects for 4 SNPs (Table 9). After correcting for multiple testing, only linear effects of *CPSF1, DEPDC7, FAM5C, MS4A8B,* and *SREBF1* were significant.

**Table 7:** **SNPs associated with milk yield**^**a**^

**SNP**	**Gene**	**Least-squares means (SEM)**			**Linear**			**Dominance**	
		**0**	**1**	**2**	**Effect**	***P *value**	**Q value**	***P *value**	**Q value**
rs110127056	*ASL*	129.19 (93.50)	76.85 (65.52)	−110.46 (78.95)	−125.97	0.0325	0.1316	0.4235	0.6041
rs43114141	*AVP*	−219.62 (96.69)	28.79 (76.21)	119.38 (80.43)	163.02	0.0070	0.0655	0.4035	0.6041
rs109032590	*BOLA-DMB*	−196.31 (132.92)	−48.42 (72.66)	84.09 (63.57)	137.68	0.0348	0.1316	0.9396	0.6799
rs135744058	*CACNA1D*	−91.04 (169.91)	−118.53 (71.28)	125.13 (61.36)	177.10	0.0103	0.0785	0.2251	0.5234
rs137601357	*CAST*	194.98 (100.40)	50.25 (63.19)	−101.42 (76.03)	−148.69	0.0150	0.0922	0.9681	0.6799
rs109621328	*CD14*	−1561.65 (401.52)	−111.12 (117.92)	87.57 (47.23)	365.61	0.0010	0.0165	0.0071	0.4733
rs134432442	*CPSF1*	−415.14 (178.65)	−181.24 (75.87)	170.79 (59.25)	323.52	<0.0001	0.0059	0.6155	0.6557
rs110270752	*DEPDC7*	166.55 (183.04)	144.62 (81.13)	−83.06 (57.35)	−175.37	0.0171	0.0933	0.0498	0.5180
rs109503725	*DSC2*	137.43 (81.39)	−34.35 (61.57)	126.23 (80.26)	−4.14	0.9427	0.6564	0.0498	0.5180
rs42075611	*DTX2*	419.42	268.38	−5.14	−235.63	0.0292	0.1274	0.7702	0.6642
		(264.53)	(162.59)	(53.42)					
rs133175991	*DZIP3*	−468.80 (238.09)	−87.50 (84.96)	114.47 (60.92)	236.29	0.0041	0.0537	0.5411	0.6557
rs135071345	*FAM5C*	91.43 (282.11)	273.07 (82.89)	−85.79 (54.42)	−281.47	0.0007	0.0153	0.0993	0.5234
rs42339105	*GOLGA4*	1083.08 (686.77)	−215.11 (108.30)	19.03 (53.55)	159.55	0.1543	0.3156	0.0341	0.5180
rs110828053	*HSD17B7*	−131.10 (2096.70)	−142.60 (79.17)	93.43 (55.37)	183.95	0.0155	0.0922	0.3517	0.6041
rs110789098	*IBSP*	−33.11 (96.27)	134.44 (70.23)	−68.21 (78.36)	−36.41	0.5439	0.5221	0.0406	0.5180
rs134011564	*MARVELD1*	1364.97 (686.88)	26.81 (52.94)	117.22 (122.41)	34.81	0.7874	0.6199	0.0432	0.5180
rs109761676	*MS4A8B*	20.93 (168.91)	161.86 (71.07)	−101.57 (63.97)	−159.13	0.0260	0.1216	0.0703	0.5180
rs109383758	*NLRP9*	−125.31 (86.14)	4.68 (64.67)	171.33 (84.75)	148.48	0.0108	0.0785	0.8290	0.6642
rs111027720	*PARM1*	−119.34 (90.81)	−35.61 (63.47)	272.73 (77.68)	204.66	0.0005	0.0153	0.1875	0.5234
rs109506766	*PGR*	−180.89 (117.82)	−39.99 (66.65)	150.10 (69.77)	172.17	0.0059	0.0644	0.7877	0.6642
rs110805802	*TDRKH*	77.83 (317.74)	142.85 (96.21)	−96.03 (53.67)	−194.34	0.0362	0.1316	0.4145	0.6041
rs132789482	*TSHB*	399.97 (280.14)	219.67 (102.03)	0.15 (56.95)	−211.99	0.0240	0.1208	0.9107	0.6799
rs134031231	*TXN2*	−37.85 (102.08)	122.46 (63.64)	−76.26 (77.60)	−44.81	0.4682	0.4715	0.0420	0.5180
rs134031231	*TXN2*	−37.85 (102.08)	122.46 (63.64)	−76.26 (77.60)	−44.81	0.4682	0.4715	0.0420	0.5180

^a^Single nucleotide polymorphism represented as the rs number given by the National Center for Biotechnology Information database SNP.

**Table 8:** **SNPs associated with fat yield**^**a**^

**SNP**	**Gene**	**Least-squares means (SEM)**			**Linear**			**Dominance**	
		**0**	**1**	**2**	**Effect**	***P *value**	**Q value**	***P *value**	**Q value**
rs41766835	*APBB1*	−0.06 (7.57)	6.22 (3.16)	12.64 (2.20)	6.39	0.0259	0.1474	0.9883	0.9719
rs110541595	*ARL6IP1*	3.52 (4.03)	5.83 (2.53)	12.47 (2.80)	4.88	0.0341	0.1516	0.5205	0.9719
rs43114141	*AVP*	3.31 (3.56)	6.03 (2.82)	12.53 (2.97)	4.76	0.0291	0.1474	0.5786	0.9719
rs133674837	*BDH2*	7.70 (4.15)	3.71 (2.57)	13.03 (2.74)	4.18	0.643	0.2443	0.0431	0.5703
rs137601357	*CAST*	18.50 (3.70)	8.56 (2.48)	4.87 (2.89)	−6.38	0.0033	0.0587	0.3021	0.9719
rs109621328	*CD14*	−15.29 (14.91)	−0.36 (4.49)	10.42 (1.94)	11.34	0.0055	0.0733	0.8097	0.9719
rs41711496	*CD40*	3.72 (3.20)	7.94 (2.48)	12.40 (3.17)	4.34	0.0479	0.1965	0.9705	0.9719
rs134432442	*CPSF1*	37.64 (6.52)	19.88 (2.79)	−0.39 (2.19)	−19.67	<0.0001	0.0048	0.7696	0.9719
rs133449166	*CSNK1E*	15.18 (3.92)	9.75 (2.57)	4.72 (2.85)	−5.19	0.0216	0.1474	0.9518	0.9719
rs110629231	*DNAH11*	−1.91 (4.66)	7.78 (2.61)	10.96 (2.55)	5.40	0.0197	0.1474	0.3446	0.9719
rs133175991	*DZIP3*	−2.51 (8.81)	5.65 (3.21)	11.90 (2.36)	6.61	0.0290	0.1474	0.8583	0.9719
FGF2ag	*FGF2*	1.05 (4.24)	7.56 (2.58)	11.43 (2.61)	4.87	0.0304	0.1474	0.6946	0.9719
rs109247499	*FST*	11.59 (3.27)	10.96 (2.50)	1.75 (3.25)	−4.94	0.0265	0.1474	0.1831	0.8095
rs43703916	*MRPL48*	0.50 (3.43)	12.51 (2.48)	6.04 (3.33)	2.59	0.2581	0.3471	0.0042	0.3149
rs111027720	*PARM1*	5.09 (3.45)	4.84 (2.48)	18.09 (2.97)	7.01	0.0014	0.0373	0.0330	0.5651
rs136457441	*RPL26*	15.50 (3.55)	6.36 (2.43)	13.19 (3.35)	−0.84	0.7208	0.5003	0.0136	0.3149
rs43321188	*SERPINE2*	−5.08 (5.73)	11.63 (2.75)	8.10 (2.27)	2.24	0.3735	0.3831	0.0118	0.3149
rs110365063	*SLC18A2*	−4.30 (5.73)	14.21 (2.75)	7.25 (2.27)	2.98	0.3407	0.3634	0.0366	0.5651
rs134031231	*TXN2*	3.94 (3.80)	13.18 (2.38)	5.89 (2.90)	−0.21	0.9263	0.5256	0.0119	0.3149

^a^Single nucleotide polymorphism represented as the rs number given by the National Center for Biotechnology Information database SNP. For entries not beginning with rs, the abbreviation given by previous researchers was used.

**Table 9:** **SNPs associated with fat percent**^**a**^

**SNP**	**Gene**	**Least-squares means (SEM)**			**Linear**			**Dominance**	
		**0**	**1**	**2**	**Effect**	***P *value**	**Q value**	***P *value**	**Q value**
rs109621328	*CD14*	0.177 (0.051)	0.016 (0.015)	0.029 (0.006)	−0.010	0.4151	0.1531	0.0033	0.1760
rs133747802	*CD2*	0.009 (0.010)	0.037 (0.017)	0.037 (0.008)	0.014	0.0348	0.0870	0.9223	0.9719
rs134432442	*CPSF1*	0.207 (0.019)	0.105 (0.008)	−0.025 (0.006)	−0.123	0.0001	0.0018	0.2690	0.4658
rs110270752	*DEPDC7*	−0.003 (0.023)	0.007 (0.010)	0.046 (0.007)	0.033	0.0004	0.0040	0.3477	0.4920
rs135071345	*FAM5C*	0.039 (0.036)	0.001 (0.011)	0.042 (0.007)	0.030	0.0053	0.0285	0.0616	0.4480
rs43079452	*HSD17B3*	0.040 (0.040)	0.055 (0.011)	0.023 (0.007)	−0.025	0.0208	0.0693	0.4153	0.9719
HSP70C895D	*HSP70*	0.020 (0.019)	0.051 (0.009)	0.020 (0.008)	−0.014	0.1009	0.1185	0.0174	0.3413
rs109761676	*MS4A8B*	0.036 (0.021)	0.012 (0.009)	0.051 (0.008)	0.023	0.0116	0.0464	0.0256	0.3413
rs134264563	*OCLN*	0.050 (0.019)	0.038 (0.009)	0.019 (0.008)	−0.017	0.0468	0.0968	0.5628	0.9719
rs41912290	*SREBF1*	0.053 (0.012)	0.035 (0.009)	0.012 (0.009)	−0.021	0.0057	0.0285	0.8490	0.5196
rs110805802	*TDRKH*	0.028 (0.041)	0.007 (0.012)	0.038 (0.007)	0.023	0.0484	0.0968	0.9191	0.9719
rs132789482	*TSHB*	0.007 (0.037)	0.007 (0.014)	0.040 (0.008)	0.014	0.0265	0.0757	0.6093	0.9719
rs137248155	*VCAN*	−0.011 (0.018)	0.040 (0.009)	0.032 (0.008)	0.010	0.1928	0.1441	0.0249	0.3413

^a^Single nucleotide polymorphism represented as the rs number given by the National Center for Biotechnology Information database SNP. For entries not beginning with rs, the abbreviation given by previous researchers was used.

For PY, there were allele substitution effects for 17 SNPs and dominance effects for 4 SNPs (Table 10). None of the effects were significant after correcting for multiple testing. For PPC, there were linear effects of 21 SNPs and 1 SNP with a dominance effect (Table 11). After correcting for multiple testing, only the linear effects of *BSP3, CPSF1, FAM5C, FCER1G, FUT1, HSPA1A, MS4A8B, PARM1,* and *TDRKH* were significant.

**Table 10:** **SNPs associated with protein yield**^**a**^

**SNP**	**Gene**	**Least-squares means (SEM)**			**Linear**			**Dominance**	
		**0**	**1**	**2**	**Effect**	***P *value**	**Q value**	***P *value**	**Q value**
rs43114141	*AVP*	−3.77 (2.96)	2.87 (2.35)	5.42 (2.48)	4.42	0.0144	0.0800	0.4680	0.7313
rs137601357	*CAST*	9.16 (3.09)	5.14 (2.00)	−1.52 (2.37)	−5.53	0.0028	0.0575	0.6131	0.7424
rs109621328	*CD14*	−39.70 (12.16)	−0.10 (3.65)	5.04 (1.58)	9.79	0.0035	0.0575	0.0146	0.3650
rs41857027	*CFDP2*	12.23 (4.90)	−1.35 (3.47)	4.31 (1.89)	−1.17	0.6037	0.4223	0.0209	0.3919
rs133449166	*CSNK1E*	10.22 (3.21)	4.36 (2.12)	−0.42 (2.35)	−5.23	0.0046	0.0575	0.8397	0.7524
rs133175991	*DZIP3*	−4.58 (7.33)	−0.03 (2.67)	6.11 (1.96)	5.84	0.0206	0.0942	0.8601	0.7589
rs43676052	*EPAS1*	−2.57 (6.94)	0.11 (2.28)	5.63 (1.83)	5.02	0.0307	0.1171	0.7324	0.7424
rs135071345	*FAM5C*	4.42 (8.71)	7.84 (2.59)	1.09 (1.72)	−5.28	0.0383	0.1226	0.3144	0.7275
rs109247499	*FST*	6.97 (2.66)	5.29 (2.03)	−3.76 (2.64)	−5.37	0.0033	0.0575	0.1623	0.6407
rs109830880	*GCNT3*	14.32 (12.06)	10.53 (3.41)	1.60 (1.99)	−8.26	0.0126	0.0788	0.7110	0.7424
rs110828053	*HSD17B7*	−2.55 (6.34)	−2.60 (2.45)	5.19 (1.78)	6.14	0.0073	0.0729	0.3258	0.7275
rs133497176	*NFKBIL1*	−16.34 (8.03)	9.12 (2.91)	2.02 (1.67)	−1.10	0.6802	0.4316	0.0010	0.0750
rs109383758	*NLRP9*	0.15 (2.64)	1.30 (2.01)	8.03 (2.60)	3.97	0.0226	0.0942	0.2734	0.7275
rs111027720	*PARM1*	1.28 (2.84)	1.13 (2.04)	8.95 (2.45)	4.15	0.0211	0.0942	0.1269	0.6309
rs109506766	*PGR*	−4.20 (3.59)	2.38 (2.06)	6.25 (2.15)	4.85	0.0102	0.0729	0.6220	0.7424
rs109629628	*PMM2*	10.21 (3.60)	4.58 (2.09)	0.05 (2.29)	−4.94	0.0095	0.0729	0.8427	0.7524
rs43572154	*ROR2*	−6.90 (9.46)	−1.15 (2.61)	4.30 (1.72)	5.49	0.0328	0.1171	0.9776	0.7789
rs43321188	*SERPINE2*	−5.56 (4.72)	2.32 (2.29)	5.06 (1.90)	4.21	0.0413	0.1226	0.4365	0.7275
rs134031231	*TXN2*	3.77 (3.14)	7.53 (2.00)	−1.97 (2.41)	−3.81	0.0417	0.12264	0.0135	0.3650

^a^Single nucleotide polymorphism represented as the rs number given by the National Center for Biotechnology Information database SNP.

**Table 11:** **SNPs associated with protein percent**^**a**^

**SNP**	**Gene**	**Least-squares means (SEM)**			**Linear**			**Dominance**	
		**0**	**1**	**2**	**Effect**	***P *value**	**Q value**	***P *value**	**Q value**
rs109967779	*ACAT2*	0.018 (0.006)	0.016 (0.004)	0.006 (0.004)	−0.007	0.0495	0.1650	0.3982	0.3071
rs110217852	*BSP3*	−0.005 (0.008)	0.009 (0.004)	0.020 (0.003)	0.022	0.0012	0.0222	0.7556	0.3129
rs109332658	*C7H19orf60*	0.005 (0.009)	0.009 (0.004)	0.018 (0.003)	0.008	0.0442	0.1628	0.6785	0.3129
rs135744058	*CACNA1D*	0.012 (0.010)	0.020 (0.004)	0.007 (0.003)	−0.008	0.0411	0.1628	0.0915	0.3040
rs109447102	*CCDC86*	0.005 (0.012)	0.006 (0.005)	0.017 (0.003)	0.009	0.0322	0.1507	0.5579	0.3129
rs134432442	*CPSF1*	0.048 (0.010)	0.027 (0.004)	0.001 (0.003)	−0.025	<0.0001	0.0063	0.7109	0.3129
rs110270752	*DEPDC7*	−0.013 (0.010)	0.012 (0.004)	0.015 (0.003)	0.009	0.0323	0.1507	0.1169	0.3040
rs109561866	*DYRK3*	−0.014 (0.023)	0.002 (0.005)	0.016 (0.003)	0.013	0.0143	0.1001	0.9134	0.3215
rs133175991	*DZIP3*	0.040 (0.013)	0.018 (0.005)	0.010 (0.003)	−0.011	0.0201	0.1109	0.4166	0.3071
rs135071345	*FAM5C*	0.007 (0.016)	−0.002 (0.005)	0.018 (0.003)	0.016	0.0006	0.0210	0.1168	0.3040
rs109137982	*FCER1G*	−0.001 (0.033)	−0.002 (0.006)	0.016 (0.003)	0.016	0.0056	0.0490	0.6054	0.3129
rs109247499	*FST*	0.022 (0.005)	0.011 (0.004)	0.006 (0.005)	−0.008	0.0163	0.1037	0.4741	0.3129
rs41893756	*FUT1*	−0.010 (0.010)	0.007 (0.005)	0.017 (0.003)	0.012	0.0046	0.0460	0.6355	0.3129
rs109262355	*FYB*	0.009 (0.006)	0.007 (0.004)	0.020 (0.004)	0.007	0.0423	0.1628	0.1740	0.3040
rs43079452	*HSD17B3*	−0.008 (0.017)	0.021 (0.005)	0.011 (0.003)	−0.005	0.2902	0.4731	0.0488	0.3040
HSP70C895D	*HSPA1A*	0.019 (0.008)	0.023 (0.004)	0.005 (0.003)	−0.012	0.0016	0.0222	0.0534	0.3040
rs109761676	*MS4A8B*	0.001 (0.009)	0.005 (0.004)	0.021 (0.004)	0.013	0.0014	0.0222	0.3892	0.3071
rs111027720	*PARM1*	0.023 (0.005)	0.013 (0.003)	0.002 (0.004)	−0.010	0.0019	0.0222	0.8492	0.3129
rs109629628	*PMM2*	0.029 (0.007)	0.011 (0.004)	0.009 (0.004)	−0.008	0.0206	0.1109	0.1396	0.3040
rs43572154	*ROR2*	0.000 (0.018)	0.005 (0.005)	0.016 (0.003)	0.010	0.0481	0.1650	0.8169	0.3129
rs41912290	*SREBF1*	0.019 (0.006)	0.015 (0.004)	0.006 (0.004)	−0.007	0.0378	0.1628	0.6750	0.3129
rs110805802	*TDRKH*	−0.001 (0.017)	0.001 (0.005)	0.017 (0.003)	0.014	0.0064	0.0498	0.5183	0.3129

^a^Single nucleotide polymorphism represented as the rs number given by the National Center for Biotechnology Information database SNP. For entries not beginning with rs, the abbreviation given by previous researchers was used.

Results for SCS are shown in Table 12. There were allele substitution effects of 8 SNPs and dominance effects for 6 SNPs. After correcting for multiple testing, the linear effects of *CFDP2, CPSF1, DSC2, FST, PMM2, SEC14L1, TXN2* and the dominance effect of *NFKBIL1* were significant.

**Table 12:** **SNPs associated with somatic cell score**^**a**^

**SNP**	**Gene**	**Least-squares means (SEM)**			**Linear**			**Dominance**	
		**0**	**1**	**2**	**Effect**	***P *value**	**Q value**	***P *value**	**Q value**
rs110127056	*ASL*	4.36 (0.16)	3.87 (0.11)	4.04 (0.14)	−0.13	0.2141	0.5831	0.0305	0.3684
rs133674837	*BDH2*	3.81 (0.19)	4.19 (0.11)	3.92 (0.12)	−0.01	0.8913	0.7535	0.0403	0.3993
rs109332658	*C7H19orf60*	4.65 (0.28)	3.89 (0.12)	4.00 (0.11)	−0.13	0.2968	0.5831	0.0234	0.3684
rs133747802	*CD2*	3.99 (0.15)	4.66 (0.25)	3.92 (0.12)	−0.28	0.0281	0.0281	0.3365	0.5851
rs41857027	*CFDP2*	4.31 (0.28)	4.34 (0.20)	3.89 (0.10)	0.26	0.0394	0.0394	0.6166	0.5851
rs134432442	*CPSF1*	3.73 (0.32)	3.83 (0.13)	4.14 (0.10)	0.26	0.0394	0.0394	0.6166	0.5851
rs109503725	*DSC2*	3.80 (0.14)	3.88 (0.11)	4.48 (0.14)	0.34	0.0008	0.0008	0.0846	0.3993
rs109247499	*FST*	3.90 (0.14)	3.89 (0.11)	4.41 (0.14)	0.25	0.0083	0.0083	0.0759	0.3993
rs133497176	*NFKBIL1*	6.52 (0.45)	3.91 (0.16)	3.97 (0.09)	−0.42	0.0030	0.1167	0.0001	0.0054
rs109629628	*PMM2*	3.84 (0.20)	3.90 (0.11)	4.24 (0.12)	0.23	0.0359	0.0359	0.4032	0.5851
rs136746215	*SEC14L1*	4.19 (0.11)	3.99 (0.14)	3.88 (0.08)	−0.15	0.0183	0.0183	0.7550	0.5851
rs132789482	*TSHB*	4.49 (0.30)	3.81 (0.11)	3.95 (0.06)	−0.02	0.8513	0.7535	0.0307	0.3684
rs134031231	*TXN2*	3.83 (0.17)	3.94 (0.10)	4.26 (0.13)	0.23	0.0352	0.0352	0.5155	0.5851

^a^Single nucleotide polymorphism represented as the rs number given by the National Center for Biotechnology Information database SNP.

### Relationship between allele substitution effects for SNPs related to DPR with effects on other traits

**Figure 1:**
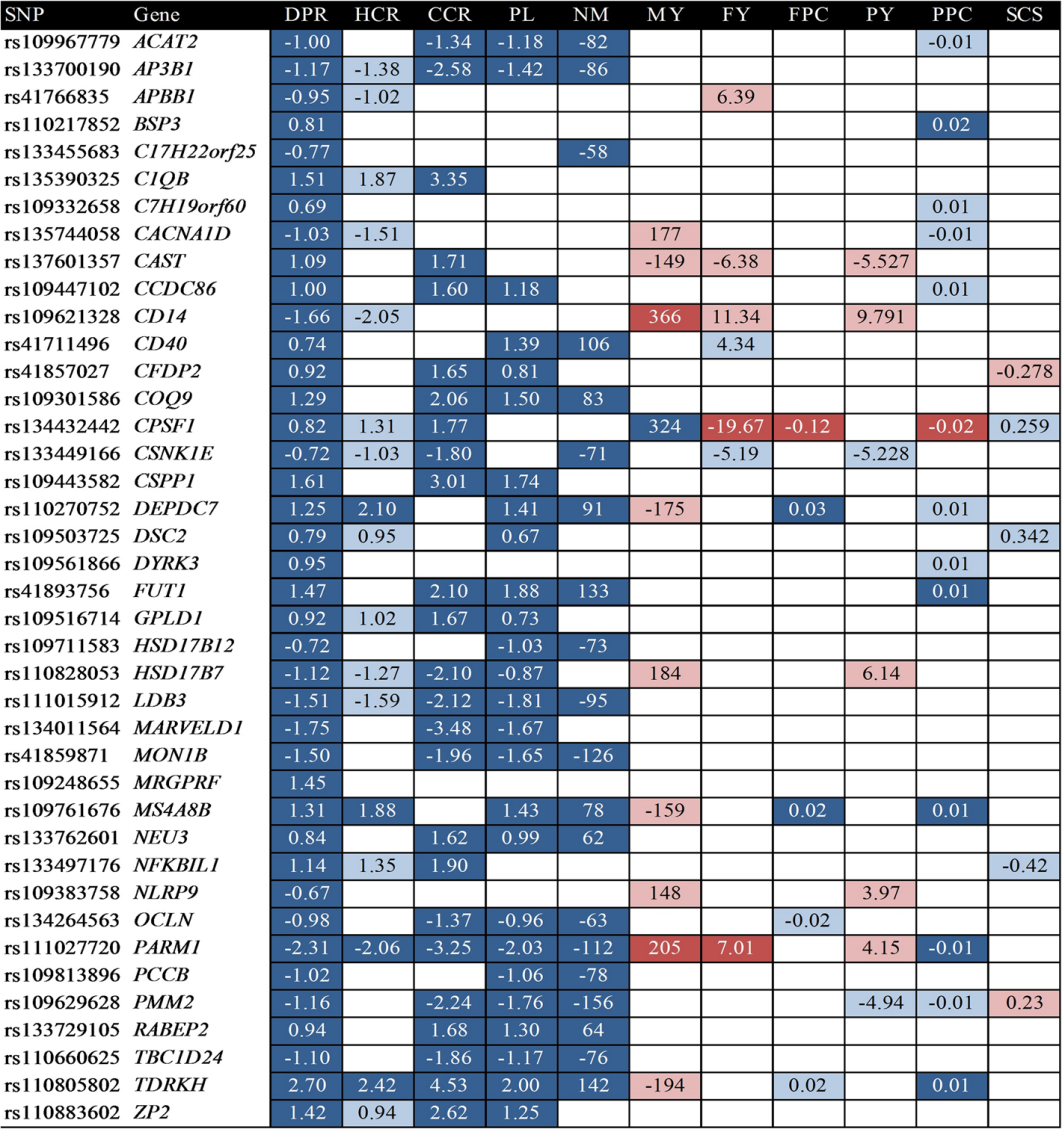
**Allele substitution effects of SNPs on fertility and production traits.** Only effects with *P* values and/or Q values < 0.05 are included. Numbers represent the magnitude of the allele substitution effect. Dark blue rectangles are linear effects in the same direction as DPR (Q < 0.05), light blue rectangles are linear effects in the same direction as DPR (*P* < 0.05 but Q value ≥ 0.05), red rectangles are linear effects in the opposite direction as DPR (*P* and Q value < 0.05), and pink rectangles are linear effects in the opposite direction as DPR (*P* < 0.05 but Q ≥ 0.05). Empty cells indicate that there was no significant effect of the SNP on the trait based on *P* or Q values. Abbreviations are as follows: CCR, cow conception rate; DPR, daughter pregnancy rate; FPC, fat percent; FY, fat yield; HCR, heifer conception rate; MY, milk yield; NM, net merit; PL, productive life; PPC, protein percent; PY, protein yield; SCS, somatic cell score.

It was determined whether SNPs affecting DPR had association with other traits and, if so, whether the allele substitution effect was in the same or opposite direction as for DPR. Results are shown in Figure 1. As expected, many SNPs associated with DPR were also associated with HCR and CCR and in the same direction as for DPR. Of 40 SNPs in which there was a linear effect on DPR (Q < 0.05), 13 also were associated with HCR and 25 were associated with CCR. In all cases, the allele substitution effect was in the same direction for DPR and either HCR or CCR. Similar results were observed for PL and NM. Of the 40 SNPs associated with DPR, 26 were also associated with PL and 20 with NM and the allele substitution effect was in the same direction for DPR and either PL and NM.

Fewer SNPs associated with DPR were also associated with production traits. Furthermore, when occurring, the direction of the effect was often in the opposite direction as for DPR, especially for yield traits. There were 10 SNPs associated with MY and all but one (*CPSF1*) were in the opposite direction as for DPR (i.e., genotypes favoring DPR were unfavorable for milk yield). There were 7 SNPs associated with FY and 5 of these were in the opposite direction as for DPR. There were 7 SNPs associated with PY and 5 of these were in the opposite direction as for DPR. For other production traits, however, there were fewer negative relationships between allele substitution effects on DPR. For FPC, there were 5 SNPs but only 1 was in the opposite direction as DPR. For PPC, there were 13 SNPs but only 1 was in the opposite direction as DPR. For SCS, there were 5 SNPs with three in the same direction as DPR and two in the opposite direction.

Of the 40 SNPs related to DPR, there were 29 that were not negatively associated with yield traits (milk, fat and protein). Thus, it should be possible to select for specific SNPs affecting DPR without compromising yield traits.

### Relationship between SNPs associated with DPR and SNPs reported previously to be related to fertility

Of the 434 SNPs analyzed, 17 were chosen because they had previously been reported to be associated with reproduction or to be close to SNPs related to interval to insemination or 56-d non-return rate (Additional file 1: Table S2). Of these, only 8 had a MAF ≥ 5% (*CAST, FGF2, FSHR, HSPA1A, IRF9, NLRP9, PGR* and *STAT5A*) and only 2 (*CAST* and *NLRP9*) were significantly associated with DPR (Table 2).

**Figure 2:**
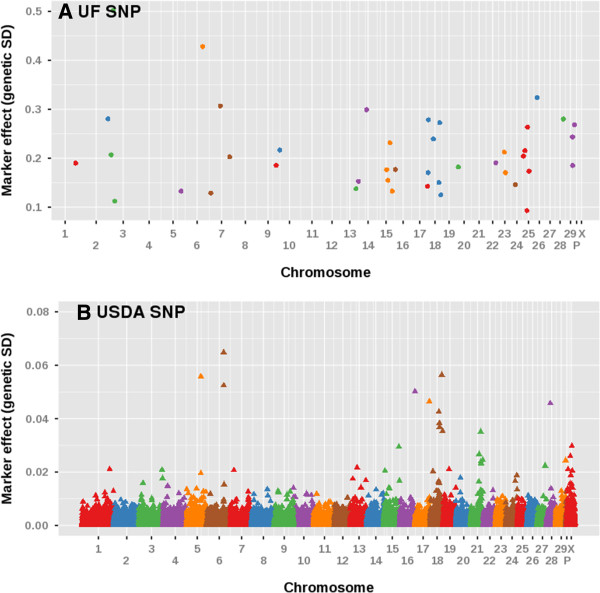
**Manhattan plots comparing SNP effects on daughter pregnancy rate from the current study (panel A; UF SNP) to marker effects from the BovineSNP50 chip in a previous study (panel B; USDA SNP) [**[7]**].** Each chromosome is represented in a different color along the x-axis. The y-axis is the marker effect on daughter pregnancy rate (genetic standard deviations). The markers are color coordinated according to their chromosome location.

The physical location of each SNP associated with DPR in the current study was compared to markers from the BovineSNP50 chip previously associated with DPR [7]. Figure 2 shows the relative location of the SNPs and the SNP50 marker effects. The SNP effects from the current custom array have a much greater effect on DPR than those found on the BovineSNP50 chip. The largest genetic standard deviation on the BovineSNP50 chip for DPR was 0.07 genetic standard deviations [7]; however, in the current study, the marker effect ranged from 0.44 to 1.78 (Additional file 3: Table S7).

A literature search was conducted to determine if any SNPs previously related to fertility were within 100,000 bases of any of the SNPs related to DPR in the current study. The literature provided evidence for 3 other SNPs located close to SNPs from the current study. A SNP in *DGAT1*, which is about 65,000 bp from the SNP in *CPSF1*, was associated with 28 and 56 day nonreturn rate to first service, age at puberty, number of inseminations per conception, and conception rate [56-58]. A SNP in *TNF*, which is about 25,000 bp from the SNP in *NFKBIL1*, was associated with early first ovulation in postpartum cows [59]. Also, a SNP in *HSD14B14*, which is about 60,000 bp from the SNP in *FUT1*, was associated with DPR [7]. Since these SNPs are close in distance, there could be linkage disequilibrium between them. Therefore, it is possible that either gene in each of the previous locations could contain the causative SNP.

### Effect of tissue type used for SNP discovery on probability of identifying SNPs associated with DPR

An analysis was performed to determine whether the tissue type used to identify genes for SNP discovery affected the probability that a gene was related to DPR (Additional file 3: Table S8). Using chi-square analysis, fewer SNPs identified in genes identified as expressed in the brain or pituitary were significantly associated with DPR (18%) than for embryo genes (51%), endometrium or oviduct genes (50%) or ovary genes (43%) (χ^2^ for brain/pituitary vs others, 3.74, *P* = 0.05).

### Pathway analysis of genes with SNPs associated with DPR

There were a total of 5 canonical pathways in which 2 or more genes were overrepresented (*P* < 0.05). These were Estrogen Biosynthesis (*HSD17B7* and *HSD17B12*), Estrogen-Dependent Breast Cancer Signaling (*HSD17B7* and *HSD17B12*), Hepatic Fibrosis/Hepatic Stellate Activation (*CD14* and *CD40*), Tight Junction Signaling (*CDSF1* and *OCLN*), and Dopamine-DARPP32 Feedback in cAMP Signaling (*CACNAID* and *CSNK1E*). The IPA software also built 4 networks of genes related to DPR. The most revealing was one that included 16 genes (*ACAT2, AP3B1, COQ9, CPSF1, CSPP1, DEPDC7, DSC2, GPLD1, HSD17B12, MARVELD1, MON1B, NFKBIL1, PMM2, RABEP2, TBC1D24,* and *TDRKH*) which interacted directly or indirectly with UBC (Additional file 3: Figure S1).

**Figure 3:**
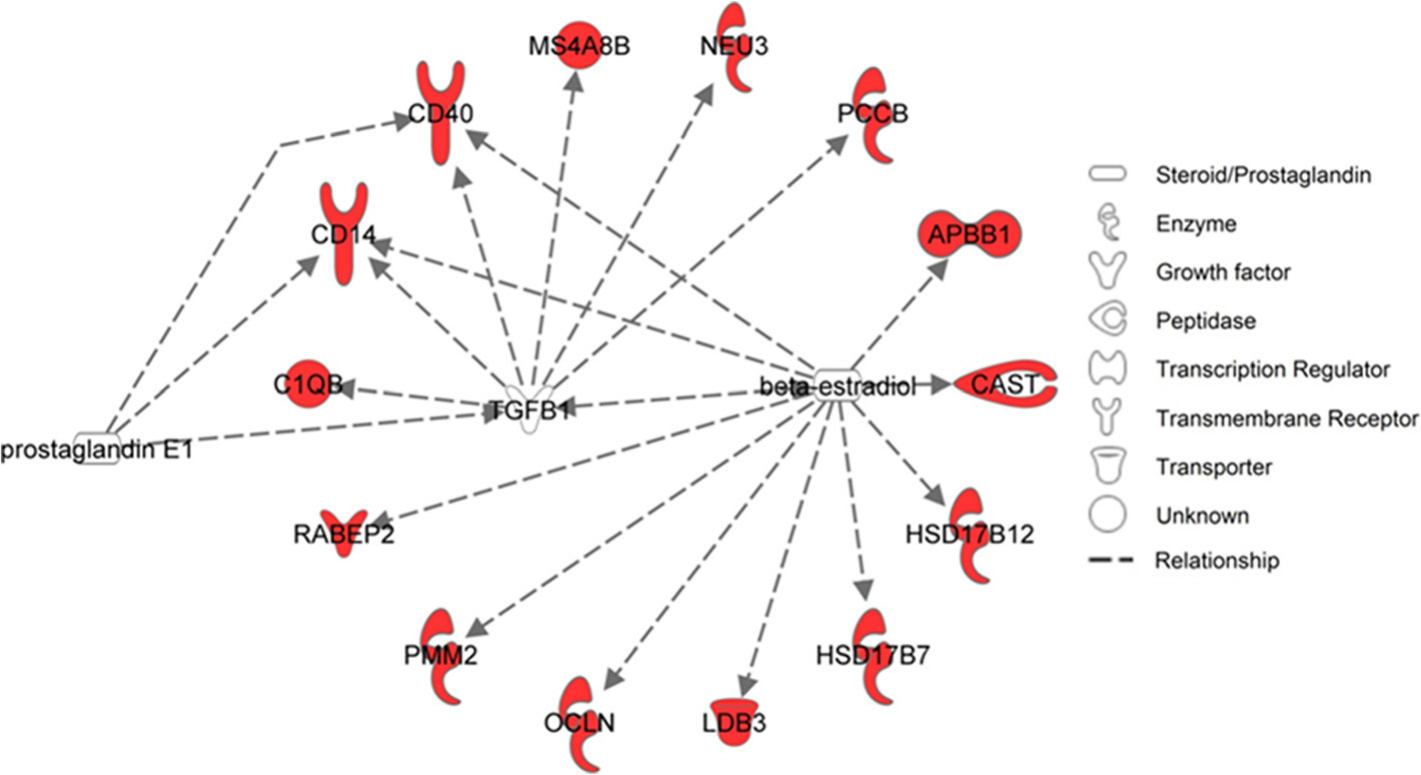
**Growth factors and steroids which regulate daughter pregnancy rate genes.** Only significant pathways are shown (*P* < 0.05). Red symbols are genes in which SNPs were associated with daughter pregnancy rate and arrows represent regulation.

The list of genes related to DPR was also examined for upstream regulators in which regulated genes were significantly (*P* < 0.05) overrepresented. A total of 5 transcription factors were identified (Additional file 3: Figure S2) including HNF4A, which regulates 8 genes associated with DPR*,* TCF3, which regulates 3 DPR genes*,* and CTBP2, FOSB, and SP100, which each regulate one gene. Additional regulators of genes associated with DPR were two hormones (estradiol and prostaglandin E1) and one growth factor (TGFB1). Estradiol regulates 10 DPR genes, TGFB1 regulates 6 genes, and prostaglandin E1 regulates 2 genes (Figure 3).

## Discussion

The results of this study verified that the candidate gene approach could be a successful method of determining markers for DPR. It was anticipated that, since the SNPs used for genotyping were specifically chosen for their function in reproductive processes, a larger proportion of them would be associated with reproductive traits than for production traits. Such a result was obtained. Of the 98 genes that met the criteria for analysis (MAF ≥ 5% and call rate ≥ 70%) and where effects were *P* < 0.05, there were 42 genes associated with DPR (Table 2) but only 23 associated with MY (Table 7). Moreover, all of the significant SNP effects for DPR in this study were between 5 and 25 times greater than the largest marker effect from the BovineSNP50 chip [7] (Figure 2 and Additional file 3: Table S7). This result is probably due to the differences in SNP selection between the two methods. The majority of SNPs on the BovineSNP50 chip are between genes (63%) and over 14,000 genes are not represented by a SNP on the Bovine SNP50 chip [9]. In the current study, almost all of the SNPs examined were located within the coding region of the gene and the remainder were close physically to the coding region. Moreover, SNPs were chosen to maximize the probability that there would be a change in the characteristic of the protein encoded for the gene. Thus, it is likely that many of the SNPs that have large effects on DPR do so because they are causative SNPs resulting in changes in protein function. The remainder may represent linkages to causative SNPs. The SNPs identified in this study may be closer to the causative SNPs than the SNPs on the BovineSNP50 chip. Allele substitution effects were estimated individually with a linear mixed model, rather than simultaneously as described in Cole et al. [7], which also could explain some of the differences.

Polymorphisms in the current study were chosen for having the greatest probability of changing protein function. In order to maximize the possibility of finding causative SNPs, we prioritized the selection of SNPs within a gene to favor those causing the greatest change in protein function. This decision may have been one reason why there was a high rate (75%) of SNPs with MAF < 5% because the SNP would be subjected to purifying selection. Only 20% of the nonsense, 25% of the missense and 9% of the frameshift mutations had MAF ≥ 5% whereas this frequency was 80% of the 5 SNPs that were in a non-coding region or did not result in an amino acid substitution. Many of the SNPs were not in Hardy-Weinberg equilibrium and this, too, may reflect the effect of the SNPs on protein function.

Of the 9 SNPs most out of equilibrium, only 3 (*CCT8*, *MARVELD1* and *SYTL2*) had less than expected frequencies of minor allele homozygotes. The interpretation is that few of the mutations in which MAF was ≥ 5% were lethal. Interestingly, for six genes, the heterozygote was more or less frequent than expected. Some of the decrease in heterozygosity could be due to inbreeding, which is high in Holstein cattle [60]. Other changes in heterozygosity could be due to either an advantage or disadvantage of the heterozygote. Heterozygote advantage could be due to the ability of receptors to recognize more forms of the peptides they bind (e.g. MHC class I [61]), heterozygotes having the optimal level of gene expression [62], or in theory, the optimal allele being different for different cell types. A reason for heterozygote disadvantage is not clear.

The antagonistic genetic relationship between fertility traits and milk production [1-3] was verified here. There was a negative correlation between DPR and MY across DPR classes (Additional file 2: Table S4) and within cows in the high DPR class (Additional file 2: Table S5). Nonetheless, there were many SNP related to DPR (and often, other reproductive and health traits) that were not antagonistic for MY. Accordingly, it should be possible to select for DPR without reducing MY. Of the 40 SNPs linearly related to DPR, only 11 were negatively associated with MY, FY, or PY (Figure 1).

SNPs that affected DPR were also positively related with other fertility traits (HCR, CCR, and NM). Other studies have also shown a positive genetic correlation among fertility traits [3,28,63]. It is not surprising that these traits are affected by the SNPs associated with DPR. One determinant of DPR is CCR. In addition, PL depends in part on the probability of culling for reproduction. The equation to calculate NM includes DPR and PL. The fact that SNPs associated with DPR are also associated with HCR, CCR, PL and NM means that selection of genes that improve DPR are likely to improve other reproductive traits and traits that depend upon reproduction.

SNPs linked to traits in the current study that were previously linked to other traits are summarized in Table 13. Of the 17 genes with SNPs previously linked to fertility or close to SNPs related to fertility traits, 9 SNPs had MAF < 5% (*BAIAP2, GHR, LEP, IGF1, IGFBP7, ITGB5, PAPPA2, SCRN1,* and *SERPINA14*) and were not analyzed. Of the other 8, 2 were significantly associated with DPR (*CAST* and *NLRP9*) and one tended to be (*FGF2*). The exact SNP in *CAST* analyzed here was previously associated with DPR, PL, and NM [15]. A different SNP in *NLRP9* than the one studied here was associated with incidence of still birth [21]. Another gene, *FGF2,* tended to have an association with DPR (*P* = 0.08), with the AA genotype being superior to the GG genotype. Previously, the AA genotype of *FGF2* was associated with higher estimated relative conception rate in bulls [16] although, surprisingly, associated with lower *in vitro* embryo development [12]. Another SNP, in *PGR,* was previously associated with *in vitro* fertilization rate and development [14] and *in vivo* fertilization [64] and pregnancy rates [65], and while not significant (*P* = 0.16), the GG genotype was superior to the CC genotype for DPR in agreement with the superior genotype seen earlier [14,64,65]. A SNP in *FSHR* was previously associated with superovulation response [19,66] and, while not significantly associated with DPR in the current study, was associated with HCR and PL. There was no significant effect of genotype for four other SNPs in genes previously associated with reproductive traits, including *HSPA1A,* associated with calving rate in beef cattle [23], *IRF9,* which was physically close to a SNP for interval to insemination [28], and *STAT5A,* associated with *in vitro* embryo development [11] and sire conception rate [16]. Note that *HSPA1A* was significantly associated with PL and NM (Table 13) and both of these traits depend upon reproductive function.

**Table 13:** **SNPs associated with at least one trait in the current study that were previously linked to one or more other traits**^**a**^

**SNP**	**Gene symbol**	**Traits in current study**	**SNP in literature**	**Trait in literature**	**Reference**
rs137601357	*CAST*	DPR, CCR, MY, FY, PY	Different location	DPR, PL, NM, SCS	[15]
rs109621328	*CD14*	DPR, HCR, NM, MY, FY, FPC, PY	Same SNP	PY, FY (tendancy for MY)	[64]
FGF2ag	*FGF2*	NM, FY	Same SNP	ERCR (bulls)	[16]
			Same SNP	FY, FPC, SCS, PL	[13]
			Same SNP	*In vitro* embryo survival to d 7	[12]
rs43745234	*FSHR*	HCR, PL	Different location	Superovulation response	[19]
			Different location	Superovulation response	[65]
HSP70C895D	*HSPA1A*	PL, NM, FPC, PPC	Same SNP	Calving rate (beef cattle)	[23]
rs41256848	*LHCGR*	PL, NM	Same SNP	Superovulation response	[66]
			Different location	Superovulation response	[19]
rs109383758	*NLRP9*	DPR, MY, PY	Different location	Incidence of stillbirth	[21]
rs109506766	*PGR*	MY, PY	Same SNP	Fertilization rate and *in vitro* embryo survival to d 7	[14]
			Different location	Superovulation response	[67]
			Different location	Pregnancy rate	[68]

^a^Single nucleotide polymorphism represented as the rs number given by the National Center for Biotechnology Information database SNP. Abbreviations are as follows: *CCR*, cow conception rate; *DPR*, daughter pregnancy rate; *ERCR*, estimated relative conception rate; *FPC*, fat percent, *FY*, fat yield; *HCR*, heifer conception rate, *MY*, milk yield; *NM*, net merit; *PL*, productive life; *PPC*, protein percent; *PY*, protein yield; and SCS, somatic cell score.

The genes in the current study with SNPs that were associated with DPR participate in a wide range of physiological functions associated with reproductive processes. Many function in the endocrine system, either in synthesis of hormones or in cell signaling. The estrogen biosynthesis pathway was one of the pathways in which genes associated with DPR were significantly overrepresented. The gene *ACAT2* is involved in cholesterol metabolism [67], and expression of *ACAT2* in cumulus cells is increased for infertile women as compared to fertile women [68]. The gene *HSD17B12* encodes for an enzyme that converts estrone to estradiol [69]. It is also involved in the synthesis of arachidonic acid and is essential for embryo survival in mice [70]. Another gene related to DPR, *HSD17B7,* also converts estrone to estradiol [71] and is essential for *de novo* cholesterol synthesis in the fetus [72-74]. In addition to genes involved in steroid synthesis, *TSHB*, a gene which codes for the β strand of the pituitary hormone, TSH, was associated with DPR. Thyroid function, which is under the control of TSH [75], can impact reproductive function in cattle [76,77]. Some genes related to DPR may also affect release of neurotransmitters controlling hypothalamic-pituitary function. One, *AP3B1,* is involved in formation of synaptic vesicles [78], and *APBB1* controls GnRH-1 neurogenesis [79]. Another, *TBC1D24,* stimulates primary axonal arborization [80,81]. Polymorphisms in *TBC1D24* have been associated with shortened axons and epileptic seizures [80,81].

Among the DPR genes involved in cell signaling are the G protein-coupled receptors *MRGPRF* and *MS4A8B*[82], *GPLD1,* which cleaves cell surface proteins anchored by phosphatidylinositol glycans [83], the sialidase *NEU3,* which is important for insulin signaling [84], *CACNA1D,* a component of calcium channels [85], and *DSC2*, an important component of membrane rafts and cell-cell junctions [86] and which is involved in blastocoel formation [87]. Similarly, *OCLN* is a major component of tight junctions and is involved in barrier stability [88]. Another gene involved in cell-cell binding related to DPR is *PMM2,* which isomerizes mannose 6-phosphate into mannose 1-phosphate [89], which eventually is converted to GDP-fucose and used to make fucosylated glycans [90]. Fucosylated glycans serve several functions, including leukocyte-endothelial adhesion, host-microbe interactions, embryo compaction, and signal transduction [90].

One gene associated with DPR, *CSNK1E,* is involved in paracrine regulation of cell function as a positive regulator of the canonical WNT/β-catenin pathway [91,92]. The WNT pathway plays important roles in cell differentiation [93,94], preimplantation development [95], formation of the epiblast [96] and implantation [97]. Moreover, *CSNK1E* regulates circadian rhythm by controlling nuclear entry of PER1, a regulator of *CLOCK*[98]. Expression of *PER1* was associated with depth of anestrus at the start of the breeding season in beef cattle [99].

Three genes related to DPR are involved with the function of spermatozoa in the female tract. The gene *BSP3* aids in maintaining sperm motility during storage in the oviduct [100]. Protein concentrations are associated with bull fertility [101] and the mRNA is down-regulated in the endometrium of heifers which carried a pregnancy to term compared to those in which the embryo died after transfer [26]. Another gene, *CAST*, plays an important role in sperm capacitation and the acrosome reaction [102-104] and may play a role in oocyte calcium-mediated processes that occur during oocyte activation [105]. The same SNP in *CAST* found to be associated with DPR in this study was earlier associated with DPR, PL, NM and SCS [15]. The embryonic gene *ZP2* encodes for a protein that makes up part of the zona pellucida and is the location that sperm bind on the zona pellucida [106,107]. One of the genes related to DPR, *NLRP9*, is likely to play an important function in the oocyte. The gene is expressed in the oocyte, and steady-state amounts of *NLRP9* mRNA decline after fertilization and become undetectable after the maternal to zygote transition [108-110].

There is much evidence to implicate immune function in the establishment of pregnancy [111]. Seven of the genes with SNPs associated with DPR are involved in immune function. The gene *C1QB* is involved in complement activation [112], *CD14* is a co-receptor for recognition of bacteria [113], *CD40* regulates cell surface receptor signaling [114], and *NFKBIL1* regulates dendritic cell function [115]. Additionally, *MON1B* and *RABEP2* help regulate phagocytosis and endocytosis [116,117] and mutations in *FUT1* have been associated with disease resistance [118-120]. Polymorphisms in *FUT1* have also been associated with total number of piglets born [121,122] and number of piglets alive at weaning [119]. It is possible that allelic variants in these genes that are positively associated with DPR improve immune function and decrease incidence of diseases such as endometritis, metritis, and mastitis that disrupt reproduction [123-125].

Three genes related to DPR are anti-apoptotic: *ARL6IP1, DYRK3* and *PARM1I*[126-128]. Induction of apoptosis in the oocyte and associated cumulus cells is associated with reduced fertilization rate [129-131]. Two molecules that improve embryo competence for establishment of pregnancy after transfer into recipients, CSF2 and IGF1 [132,133], are anti-apoptotic in embryos [32,134].

A variety of other roles are also represented by the genes with SNPs associated with DPR. Two genes are involved in energy pathways (*COQ9* and *PCCB*). The COQ9 protein is necessary for the synthesis of CoQ10 [135], which is needed for generating ATP [136]. PCCB is an enzyme that converts proponyl CoA to methylmalonyl CoA during gluconeogenesis [137]. The *CSPP1* gene plays a role in spindle formation and cytokinesis [138], *MARVELD1* inhibits cell cycle progression and migration [139], and *LDB3* helps organize actin and α-actinin binding in sarcomeres [140]. Finally, *CPSF1* is involved in 3′ end-processing of pre-messenger RNAs into messenger RNAs [140].

Several gene networks were significant among the genes related to DPR but most contained only two genes. The exceptions were estrogen biosynthesis, discussed earlier, and a network of genes associated with ubiquitin C (UBC). It is not surprising that the proteins encoded for by so many genes bind to UBC because ubiquitin is involved in a large number of intracellular functions [141-144]. Five transcription factors (HNF4A, TCF3, CTBP2, FOSB, and SP100), two hormones (estradiol and prostaglandin E1), and one growth factor (TGFB1) were determined by the IPA software to be significantly overrepresented as regulators of DPR genes. Each of these upstream regulators could be studied further for the potential to improve fertility by regulating activation of pathways controlled by these molecules.

## Conclusions

In conclusion, SNPs in a total of 40 genes associated with DPR were identified as well as SNPs for other traits. It might be feasible to include these SNPs into genomic tests of reproduction and other traits. The genes associated with DPR are likely to be important for understanding the physiology of reproduction and manipulating reproduction function in cattle. Given the large number of SNPs associated with DPR that were not negatively associated with production traits, it should be possible to select for DPR without compromising production.

## Abbreviations

CCR: Cow conception rate; DPR: Daughter pregnancy rate; ERCR: Estimated relative conception rate; FPC: Fat percent, FY, Fat yield; HCR: Heifer conception rate; MAF: Minor allele frequency; MY: Milk yield; NM: Net merit; PL: Productive life; PPC: Protein percent; PTA: Predicted transmitting ability, PY, Protein yield; SCS: Somatic cell score; SNP: Single nucleotide polymorphism.

## Competing interests

The authors declare that they have no competing interests.

## Authors’ contributions

Conceived and designed the experiments: SDC, PJH, JBC. Performed the experiments: SDC. Analyzed the data: SDC, DJN, JBC, PJH. Wrote initial drafts of the paper: SDC, PJH. All authors read and approved the final manuscript.

## Supplementary Material

Additional file 1: Table S1 Predicted transmitting ability for selected traits of bulls used for genotyping. **Table S2.** Source of genes included in SNP array. **Table S3.** Full list of SNPs used in array. Click here for file

Additional file 2: Table S4 Correlations among predicted transmitting ability for traits on bulls used for genotyping. **Table S5.** Correlations, within DPR class, among predicted transmitting ability for traits on bulls used for genotyping. **Table S6.** Information for all SNPs with minor allele frequencies (MAF) > 5% and call rates > 70%. Click here for file

Additional file 3: Table S7 Genetic standard deviations of SNPs associated with daughter pregnancy rate. **Table S8.** Effect of tissue type in which genes were identified on the percent of genes that were significantly associated with daughter pregnancy rate (DPR). **Figure S1**. The ubiquitin pathway contains an overrepresentation of daughter pregnancy rate genes. **Figure S2.** Transcription factors which regulate daughter pregnancy rate genes. Click here for file
