# Supplementary material for: Discovery of single nucleotide polymorphisms in candidate genes associated with fertility and production traits in Holstein cattle
Source: BMC Genet. 2013 Jun 7;14:49. doi: 10.1186/1471-2156-14-49 (PMC3686577; doi:10.1186/1471-2156-14-49)
Supplement: Additional file 1: Table S1 — Predicted transmitting ability for selected traits of bulls used for genotyping. Table S2. Source of genes included in SNP array. Table S3. Full list of SNPs used in array. [file 1471-2156-14-49-S1.pdf]

Table S1. Predicted transmitting ability for selected traits of bulls used for genotyping.

Trait	Mean	Standard error	Minimum	Maximum
Daughter pregnancy rate	-0.17	3.25	-5.90	5.30
Heifer conception rate	0.15	1.78	-6.40	5.80
Cow conception rate	-0.31	4.13	-8.50	8.20
Productive life	0.43	3.91	-7.70	9.90
Net merit	47.49	347.58	-948.00	867.00
Milk yield	13.78	1036.00	-4386.00	2571.00
Fat yield	6.23	36.55	-139.00	107.00
Percent fat	0.02	0.11	-0.26	0.50
Protein yield	2.67	29.55	-121.00	62.00
Percent protein	0.01	0.05	-0.12	0.18
Somatic cell score	2.95	0.22	2.41	3.68

Table S2. Source of genes included in SNP array.

Source	Genes used for SNP analysis	Reference
Genes with SNPs previously linked to reproduction	*CAST, FGF2, FSHR, GHR, HSPA1A, LEP, ITGB5, NLRP9, PAPPA2, PGR, STAT5A, SERPINA14*	11, 12, 14, 15,17, 18, 19, 21, 22, 23, 24, 49
Genes physically close to SNPs related to interval to insemination	*IGFBP7, IRF9, PARP12*	28
Genes physically close to 56 d non-return rate	*BAIAP2, SCRN1*	28
Genes differentially regulated in the brain of animals displaying strong vs. weak estrus	*A2ML1, ATP1A2, BNIP2, CACNA1D, CALCR, CCDC137, CCDC88B, CHRNA5, CLDN11, DOK2, DLGAP1, DNAH11, ENPP2, FAM5C, FBP2, FILIP1, FXC1, GNA12, GNA15, HEPACAM, HIGD1B, HTR2A, LAT, LRRC8D, MAPK8IP3, MCHR1, MCOLN3, MOCOS, MOCS1, NKD1, PDIA5, PLP2, PLXNC1, POMC, PRKCQ, RHBDF1, RRP7A, SNX1, SNX27, SNX7, SPARCL1, SPTBN4, SYTL2, THBD THBS1, TRPV3, TSC22D2, TSPAN15, TSPYL1, TTF1, VAT1L,*	25
Embryo genes upregulated after cryopreservation	*DSC2*	29
Genes differentially regulated in superovulated embryos compared to embryos from unstimulated dams	*CLGN, GOLGA4, HNRNPUL1, HSPA14, JARID2, KIT, FUT1, MYO1D*	30
Genes differentially regulated in embryos which survived to term compared to embryos that died *in vivo*	*ACAT2, AP2B1, ATP5A1, CCT2, CCT8, DHRS12, DTX2, GART, GDE1, GPX4, HNRPA1, HSPA8, KIF20A, KPNA4, MS4A8B, NDUFS4, OCLN, PRSS23, RIOK3, RPL7A, RPL8, TAGLN2, WDR13, WDR26, ZNF207, ZNF638*	27, 31
Genes differentially regulated in oocytes compared to 8 cell embryos	*CD14, CD97, CLIC4, COPZ1, CTNNBL1, GTF2F1, IL1A, MAOA, NEU3, NR2F2, PISD, POLD2, PTGFR, TXN2, TXNL4B, UQCRC2, ZDHHC16*	35
Genes differentially regulated in oocytes compared to blastocysts	*CLIC1, GJA1, GRB10, HMGB2, HPRT, NUP160, TAF9,*	36
Genes differentially regulated embryos treated with CSF2 compared to control embryos	*ATP10D, BRD3, CACNA1G, CCRL1, CD2, CCDC86, CELSR2, CENPI, CHURC1, C17H22orf25, DTX3, DZIP3, ECE1, ECE2, F5, FGD3, FHL1, FYB, GREM1, HOXA5, HUWE1, IBSP, IMPDH1, LANCL2, LEMD2, MASP2, MASTL, MPP2, MRPS12, NT5E, PCDH24, PHGDH, PLAC8L1, PLD2, PMM2, POLL, PPP1R3F, PPP2R3A, PTPRK, RALGPS1, ROR2, RTN4, SFRP4, SNX29, STUB1, SULT2A1, TBC1D24, THBS3, THYN1, TMEM17, TMEM98, TNFSF8, TTLL3, ZNF135, ZNF180, ZNF521*	32
Genes differentially regulated in embryos treated with IGF1 compared to control embryos	*ARHGEF10L, ATP9B, C5H12orf11, COL4A1, COQ9, CPSF3, CREG1, CYP4F2, DEPDC7, DPYSL4, DYRK3, FADS6, GCNT3, GPR173, H1FOO, IMMT, IPP, MMP13, MMP15, MON1B, MST1, NEURL, OSBPL11, PGCP, RNASEL, SLC40A1, TDRKH, TRAM2*	33
Genes differentially regulated in embryos cultured *in vitro* in the well of the well system compared to embryos cultured in groups	*ACOT8, ACTB, ALOX15, ATP2B3, BUB3, CFDP2, CSNK1E, GDPD5, MNS1, MTERFD1, NUSAP1, RGS16, TKDP1, UHRF1, ZP4*	34
Endometrial genes related to embryo survival	*AGPAT4, DGKA, BSP3*	26

Table S2 (continued).

Source	Genes used for SNP analysis	Reference
Endometrial genes differentially regulated in lactating cows compared to non-lactating cows	*ABCD1, ANGPTL4, AP3D1, APBB1, AQP4, ARIH1, ARL6IP1, ATP2A2, BAG3, BCAS1, BREH1, C23H6orf145, C7H19orf60, CA5B, CCDC124, CGREF1, CHCHD6, CNN1, COMMD5, COMTD1, COPS6 CSPP1, EGFL8, EIF2AK3, EIF2C2, EIF2C3, ELAC1, FAM33A, FXR1, GALT, GRASP, HAX1, HEYL, HIPK1, HIVP1, HMCN1, HSDL2, ICA1, KCNN2, KDR, KLK1, LPIN3, MACF1, MARVELD1, MCM5, MCPH1, MDH1B, MGC143117, MRGPRF, MRPL48, MST4, NAP1L5, NDUFB10, NFKBIL1, ODF2, PAPOLG, PDK4, PDLIM4, PEMT, PEX11G, RABEP2, RAGE, SEC14L1, SLC1A5, SLC39A13, SSFA2, ST13, TALDO1, TMBIM4, TNMD, UBE2D3P, WBP1, ZNF45*	38
Endometrial genes differentially regulated in pregnant cows compared to non-pregnant cows	*ABCC3, ABHD2, ABLIM3, ADFP, AHCYL2, AIFM1, ALG14, ANXA1, ARHGAP15, ASL, ATL3, BCL2L1, BCL2L15, BOLA-DMB, C10H14orf10, C1QB, C2, C28H10orf10, CARS2, CBX6, CCDC80, CD40, CD59, CDH13, CFB, CNP, COL11A1, COL18A1, CRYM, DAXX, PARM1, DNASE2, DPP3, DRAM, EIF2AK2, ENPP3, EPAS1, EVI2B, FABP3, FAP, FBLN5, FCER1G, GALNTL1, GBP4, GBP5, GPLD1, GRIK1, GTF2IRD, PLET1*	38
Genes in cumulus cells regulated by the LH surge	*DHCR24, ERRF11, HAS2, SLC18A2*	39
Cumulus genes differentially regulated at different stages of oocyte maturation	*AP3B1, CAPN10, CLU, CSDE1, ETF1, LDB3, LDLR, PDAI3, PLA2G1B, PPIB, RPS5*	40
Genes differentially regulated in dominant follicles compared to subordinate follicles	*APBB1IP, VCAN, CYP19A1, DDX6, FAM65B, FST, MAP3K5, SERPINE2, WDR77*	42-45
Genes differentially regulated in cumulus cells from *in vivo* embryos compared to *in vitro* embryos	*LPL, MAGED1*	41
Liver genes differentially regulated during the transition period	*ACLY, ACSL1, BDH2, CPT2, GPD2, NR1H3, PC, PCCA, PCCB, PPARA, SREBF1*	46
Mammary genes differentially regulated during lactation	*ABCA1*	47
Oocyte genes differentially regulated at different stages of oocyte maturation	*CPSF1, HSF1, PAPOLA, ZP2*	37
Genes differentially regulated in oviduct of cows at diestrus compared to estrus	*C3, EDEM, NARG2, OVGP1, REPS1, RPL26, SLC35B1, SNX9*	48
Other genes of interest	*ADRB3, AVP, AVPR1B, BCL2, BMP15, CSF2, FGFR1, FGFR3, FGFR4, GDF9, GH1, HCRT, HCRTR2, HSD17B6, HSD17B10, HSD17B12, HSD17B14, HSD17B3, HSD17B7, HSPA9, IFNT2, IGF2R, INHA, INS, LEPR, LHCGR, LIF, NPY2R, POU5F1, PRLR, SPP1, TSHB*	Chosen for well-established role in reproduction

Table S3. Full list of SNPs used in array.[ab]

SNP	Gene symbol	Type	SNP	Gene symbol	Type	SNP	Gene symbol	Type
rs136237997	*A2ML1*	F	rs135769862	*C10H14orf37*	M	rs135150607	*COPZ1*	M
rs134363828	*ABCA1*	M	rs41749178	*C15H11ORF34*	M	rs109301586	*COQ9*	M
rs41909830	*ABCC3*	M	rs135390325	*C1QB*	M	rs134432442	*CPSF*	M
rs133760069	*ABCD1*	M	rs136246323	*C2*	M	rs43689571	*CPSF3*	M
rs133886423	*ABHD2*	M	rs133455683	*C22orf25*	M	rs110644120	*CPT2*	M
rs43521241	*ABLIM3*	M	rs132686349	*C23H6orf145*	M	rs136509175	*CREG1*	M
rs109967779	*ACAT2*	M	rs134150850	*C28H10ORF10*	M	rs135388644	*CRYM*	M
rs134226536	*ACLY*	M	rs137306001	*C3*	M	rs137419143	*CSDE1*	M
rs132631661	*ACOT8*	M	rs134731843	*C5H12orf11*	M	rs134570201	*CSF2*	M
rs133807533	*ACSL1*	M	rs109332658	*C7H19orf60*	M	rs133449166	*CSNK1E*	M
rs109328897	*ACTB*	M	rs134920430	*CA5B*	F	rs137248155	*CSPG2*	M
rs136470751	*ADFP*	M	rs135744058	*CACNA1D*	M	rs109443582	*CSPP1*	M
rs134342155	*ADRB3*	M	rs135633806	*CACNA1G*	M	rs135969377	*CTNNBL1*	M
rs134151532	*AGPAT4*	F	rs42940189	*CALCR*	M	rs72552260	*CYP19A1*	N
rs41599809	*AHCYL2*	M	rs135946207	*CAPN10*	M	rs135537165	*CYP4F2*	M
rs137266161	*AIFM1*	M	rs133633034	*CARS2*	M	rs136421630	*DAXX*	M
rs134553846	*ALG14*	M	rs137601357	*CAST*	M	rs134436813	*DDX6*	M
rs135335430	*ALOX15*	M	rs136677334	*CBX6*	M	rs110270752	*DEPDC7*	M
rs133045346	*ANGPTL4*	M	rs137496152	*CCDC124*	M	rs43431357	*DGKA*	M
rs133874793	*ANXA1*	M	rs109405883	*CCDC137*	M	rs43350963	*DHCR24*	M
rs109258025	*AP2B1*	M	rs135044672	*CCDC80*	M	rs136114393	*DHRS12*	M
rs133700190	*AP3B1*	M	rs109447102	*CCDC86*	M	rs111027720	*DKFZP564O0823*	M
rs109135734	*AP3D1*	M	rs41652321	*CCDC88B*	M	rs134091448	*DLGAP1*	M
rs41766835	*APBB1*	M	rs136780154	*CCRL1*	M	rs110629231	*DNAH11*	M
rs110220591	*APBB1IP*	M	rs137824263	*CCT2*	N	rs43498653	*DNASE2*	M
rs137167347	*AQP4*	M	rs137673698	*CCT8*	M	rs109455239	*DOK2*	M
rs135611444	*ARHGAP15*	N	rs109621328	*CD14*	M	rs136449785	*DPP3*	M
rs110409773	*ARHGEF10L*	M	rs133747802	*CD2*	M	rs137755291	*DPYSL4*	M
rs132929646	*ARIH1*	M	rs41711496	*CD40*	M	rs42500325	*DRAM*	M
rs110541595	*ARL6IP1*	N	rs135772848	*CD59*	M	rs109503725	*DSC2*	M
rs110127056	*ASL*	M	rs136773663	*CD97*	N	rs42075611	*DTX2*	M
rs132809107	*ATL3*	M	rs135844880	*CDH13*	M	rs42802714	*DTX3*	M
rs136026021	*ATP10D*	M	rs132778427	*CELSR2*	M	rs109561866	*DYRK3*	M
rs134914858	*ATP1A2*	M	rs42659465	*CENPI*	M	rs133175991	*DZIP3*	M
rs135566160	*ATP2A2*	M	rs110908357	*CFB*	M	rs134627657	*ECE1*	M
rs137598162	*ATP2B3*	M	rs41857027	*CFDP2*	M	rs43246430	*ECE2*	M
rs110909003	*ATP5A1*	M	rs136313735	*CGREF1*	M	rs136063807	*EDEM*	F
rs133943579	*ATP9B*	M	rs42015101	*CHCHD6*	M	rs133368470	*EGFL8*	M
rs43114141	*AVP*	M	rs133347185	*CHRNA5*	M	rs134328637	*EIF2AK2*	M
rs134002804	*AVPR1B*	M	rs134702715	*CHURC1*	M	rs132729335	*EIF2AK3*	M
rs136509758	*BAG3*	M	rs135468863	*CLDN11*	M	rs134157159	*EIF2C2*	M
rs111023026	*BAIAP2*	M	rs136761255	*CLGN*	M	rs133045474	*EIF2C3*	M
rs109669573	*BCAS1*	M	rs136412422	*CLIC1*	M	rs42048798	*ELAC1*	M
rs135348971	*BCL2*	M	rs135662651	*CLIC4*	F	rs41743834	*ENPP2*	M
rs137820357	*BCL2L1*	M	rs132831699	*CLU*	M	rs135184987	*ENPP3*	M
rs137705645	*BCL2L15*	M	rs134527215	*CNN1*	M	rs43676052	*EPAS1*	M
rs133674837	*BDH2*	M	rs135658705	*CNP*	M	rs132928554	*ERRF11*	M
rs81184812	*BMP15*	M	rs42524413	*COL11A1*	M	rs43518269	*ETF1*	M
rs42556894	*BNIP2*	M	rs136347771	*COL18A1*	M	rs137607833	*EVI2B*	M
rs109032590	*BOLA-DMB*	M	rs109203188	*COL4A1*	M	rs133157169	*F5*	F
rs137512132	*BRD3*	M	rs110745686	*COMMD5*	M	rs109296407	*FABP3*	M
rs133216336	*BREH1*	M	rs135553308	*COMTD1*	F	rs135036564	*FADS6*	M
rs136142980	*BUB3*	M	rs133636751	*COPS6*	M	rs137620068	*FAM33A*	M

Table S3 (continued).[ab]

SNP	Gene symbol	Type	SNP	Gene symbol	Type	SNP	Gene symbol	Type
rs135071345	*FAM5C*	M	rs137637327	*HIVEP1*	M	rs43362191	*MACF1*	M
rs109828652	*FAM65B*	M	rs41820800	*HMCN1*	M	rs132883208	*MAGED1*	M
rs41644070	*FAP*	M	rs137106841	*HMGB2*	M	rs134256715	*MAOA*	M
rs136311048	*FBLN5*	M	rs136846474	*HNRNPUL1*	M	rs35828944	*MAP3K5*	F
rs137041504	*FBP2*	M	rs134667792	*HNRPA1*	M	rs133744546	*MAPK8IP3*	M
rs109137982	*FCER1G*	M	rs133324316	*HOXA5*	F	rs134011564	*MARVELD1*	M
rs136479492	*FGD3*	M	rs111004439	*HPRT*	M	rs135861024	*MASP2*	M
FGF2ag	*FGF2*	I	rs137159760	*HSD17B10*	M	rs135231416	*MASTL*	M
rs136916726	*FGF2*	M	rs109711583	*HSD17B12*	M	rs110032311	*MCHR1*	M
rs135399289	*FGFR1*	M	rs136102662	*HSD17B14*	M	rs110120925	*MCM5*	M
rs135467439	*FGFR3*	M	rs43079452	*HSD17B3*	M	rs109745895	*MCOLN3*	M
rs132906456	*FGFR4*	M	rs109769865	*HSD17B6*	M	rs133189515	*MCPH1*	M
rs132951036	*FHL1*	M	rs110828053	*HSD17B7*	M	rs133974623	*MDH1B*	M
rs133920062	*FILIP1*	M	rs136004185	*HSDL2*	F	rs134555355	*MGC143117*	M
rs43745234	*FSHR*	M	rs135258919	*HSF*	M	rs41893756	*MGC152033*	M
rs109247499	*FST*	M	HSP70C895D	*HSPA1A*	P	rs111015874	*MMP13*	M
rs109417696	*FXC1*	M	rs109582484	*HSPA14*	M	rs109314167	*MMP15*	M
rs135373537	*FXR1*	M	rs135485189	*HSPA8*	M	rs135686109	*MNS1*	M
rs109262355	*FYB*	M	rs134429278	*HSPA9*	M	rs133930203	*MOCOS*	M
rs137308622	*GALNTL1*	M	rs137432044	*HTR2A*	M	rs136684662	*MOCS1*	M
rs135558719	*GALT*	M	rs134861564	*HUWE1*	M	rs41859871	*MON1B*	M
rs133161316	*GART*	M	rs110789098	*IBSP*	M	rs136665691	*MPP2*	M
rs136339580	*GBP4*	M	rs135022753	*ICA1*	M	rs109248655	*MRGPRF*	M
rs136297934	*GBP5*	M	rs133621844	*IFNT*	M	rs43703916	*MRPL48*	M
rs109830880	*GCNT3*	M	rs134815699	*IGF2R*	M	rs135290352	*MRPS12*	M
rs133381146	*GDE1*	M	rs43477921	*IGFBP7*	M	rs109761676	*MS4A8B*	M
rs110553528	*GDF9*	M	rs135971907	*IL1A*	M	rs109866722	*MST1*	M
rs133081463	*GDPD5*	M	rs135674052	*IMMT*	M	rs135493649	*MST4*	F
rs134687399	*GH1*	M	rs109755950	*IMPDH1*	M	rs134254857	*MTERFD1*	M
rs109212162	*GHR*	M	rs43322178	*INHA*	M	rs42592965	*MYO1D*	M
rs136325310	*GJA1*	M	rs109229312	*INS*	M	rs109383758	*NALP9*	M
rs132685523	*GNA12*	M	rs133441883	*IPP*	M	rs133855844	*NAP1L5*	M
rs134740563	*GNA15*	M	rs110953315	*IRF9*	M	rs43632414	*NARG2*	M
rs42339105	*GOLGA4*	M	rs137747481	*ITGB5*	M	rs109162730	*NDUFB10*	M
rs134191500	*GPD2*	M	rs136141098	*JARID2*	M	rs136596433	*NDUFS4*	M
rs109516714	*GPLD1*	M	rs135848069	*KCNN2*	M	rs133762601	*NEU3*	M
rs137471680	*GPR173*	M	rs110675167	*KDR*	M	rs135719210	*NEURL*	M
rs133501979	*GPX4*	M	rs137783797	*KIF20A*	M	rs133497176	*NFKBIL1*	M
rs136675254	*GRASP*	M	rs109770509	*KIT*	M	rs133303839	*NKD1*	M
rs109131402	*GRB10*	M	rs136160116	*KLK1*	M	rs136245753	*NPY2R*	M
rs134515406	*GREM1*	M	rs133611510	*KPNA4*	M	rs41780084	*NR1H3*	N
rs42677586	*GRIK1*	M	rs135980601	*LANCL2*	M	rs137384129	*NR2F2*	M
rs136907804	*GTF2F1*	F	rs133552701	*LAT*	M	rs42508588	*NT5E*	M
rs137543809	*GTF2IRD1*	M	rs111015912	*LDB3*	M	rs136067760	*NUP160*	F
rs43565520	*H1FOO*	M	rs137254593	*LDLR*	M	rs133025321	*NUSAP1*	M
rs137419895	*HAS2*	M	rs109640782	*LEMD2*	M	rs134264563	*OCLN*	M
rs110733329	*HAX1*	M	rs29004487	*LEP*	M	rs109118553	*ODF2*	M
rs137237970	*HCRT*	M	rs109178802	*LEPR*	N	rs134635807	*OSBPL11*	M
rs135832477	*HCRTR2*	M	rs41256848	*LHCGR*	M	rs110568464	*OVGP1*	M
rs136372325	*HEPACAM*	M	rs133270360	*LIF*	M	rs137633593	*PAPOLA*	M
rs135822443	*HEYL*	M	rs135714188	*LPIN3*	M	rs134114239	*PAPOLG*	M
rs136118679	*HIGD1B*	M	rs133043641	*LPL*	M	rs42301978	*PAPPA2*	I
rs132644376	*HIPK1*	M	rs133530005	*LRRC8D*	M	rs137606191	*PARP12*	M

Table S3 (continued).[ab]

SNP	Gene symbol	Type	SNP	Gene symbol	Type	SNP	Gene symbol	Type
rs137233944	*PC*	M	rs109899155	*RIOK3*	M	rs133088824	*THBD*	M
rs135481809	*PCCA*	M	rs133308936	*RNASEL*	M	rs34755712	*THBS1*	F
rs109813896	*PCCB*	M	rs43572154	*ROR2*	M	rs43334659	*THBS3*	M
rs137801136	*PCDH24*	M	rs136457441	*RPL26*	M	rs134923946	*THYN1*	M
rs136315768	*PDAI3*	M	rs109154196	*RPL7A*	M	rs110506989	*TKDP1*	M
rs134930259	*PDIA5*	M	rs110729074	*RPL8*	M	rs133648944	*TMBIM4*	M
rs136831664	*PDK4*	F	rs134055596	*RPS5*	F	rs136831911	*TMEM17*	M
rs135843349	*PDLIM4*	M	rs109755996	*RRP7A*	M	rs137060463	*TMEM98*	M
rs135340897	*PEMT*	M	rs137808837	*RTN4*	M	rs43565960	*TNFSF8*	M
rs133122008	*PEX11G*	M	rs137549891	*SCRN1*	M	rs135290028	*TNMD*	M
rs137033579	*PGCP*	M	rs136746215	*SEC14L1*	M	rs110918953	*TRAM2*	M
rs109506766	*PGR*	I	rs43321188	*SERPINE2*	M	rs137129346	*TRPV3*	M
rs110835307	*PHGDH*	M	rs136448399	*SFRP4*	M	rs137261498	*TSC22D2*	M
rs136897415	*PISD*	M	rs110365063	*SLC18A2*	M	rs132789482	*TSHB*	M
rs133904031	*PLA2G1B*	M	rs132897655	*SLC1A5*	F	rs133709728	*TSPAN15*	M
rs135040893	*PLAC8L1*	M	rs135216399	*SLC35B1*	M	rs43445397	*TSPYL1*	M
rs110297919	*PLD2*	N	rs136807999	*SLC39A13*	M	rs136069690	*TTF1*	M
rs135334440	*PLP2*	M	rs135002561	*SLC40A1*	M	rs135236119	*TTLL3*	M
rs135342407	*PLXNC1*	M	rs137656577	*SNX1*	M	rs134031231	*TXN2*	M
rs109629628	*PMM2*	M	rs135665173	*SNX27*	M	rs134335918	*TXNL4B*	M
rs132811001	*POLD2*	M	rs134807505	*SNX29*	M	rs137527222	*UBE2D3P*	M
rs132800212	*POLL*	M	rs135563342	*SNX7*	M	rs134818016	*UHRF1*	F
rs134398664	*POMC*	M	rs132695659	*SNX9*	F	rs43707902	*UQCRC2*	M
rs109482560	*POU5F1*	M	rs135175033	*SPARCL1*	M	rs133324345	*UTMP*	M
rs110497132	*PPARA*	M	rs133929040	*SPP1*	M	rs133485956	*VAT1L*	M
rs133714443	*PPIB*	M	rs134981429	*SPTBN4*	M	rs134282928	*WBP1*	M
rs135925432	*PPP1R3F*	M	rs41912290	*SREBF1*	M	rs133862322	*WDR13*	M
rs136046311	*PPP2R3A*	M	rs42239334	*SSFA2*	M	rs135193843	*WDR26*	F
rs135992250	*PRKCQ*	M	rs132679139	*ST13*	M	rs43332935	*WDR77*	M
rs43158737	*PRLR*	N	rs137182814	*STAT5A*	S	rs134496020	*ZDHHC16*	M
rs136084042	*PRSS23*	M	rs135050491	*STUB1*	F	rs41900150	*ZNF135*	M
rs135545931	*PTGFR*	M	rs135453446	*SULT2A1*	F	rs133736498	*ZNF180*	M
rs135033411	*PTPRK*	M	rs110217852	*SVS8*	M	rs134468746	*ZNF207*	M
rs133729105	*RABEP2*	M	rs42158454	*SYTL2*	M	rs41729882	*ZNF45*	M
rs134108418	*RAGE*	M	rs42525506	*TAF9*	M	rs137362861	*ZNF521*	M
rs135078185	*RALGPS1*	M	rs135123038	*TAGLN2*	M	rs43662395	*ZNF638*	M
rs133947085	*REPS1*	M	rs110135149	*TALDO1*	M	rs110883602	*ZP2*	M
rs137380157	*RGS16*	M	rs110660625	*TBC1D24*	N	rs133973115	*ZP4*	M
rs137192728	*RHBDF1*	M	rs110805802	*TDRKH*	M			

[a]Single nucleotide polymorphism represented as the rs number designated by the National Center for Biotechnology Information dbSNP. For entries not beginning with rs, the abbreviation given by previous researchers was used. [b]Abbreviations are: SNP, single nucleotide polymorphism; Type, type of polymorphism; F, frameshift; I, intron; N, nonsense; M, missense; P, promoter; and S, synonymous.
