# Supplementary material for: Discovery of single nucleotide polymorphisms in candidate genes associated with fertility and production traits in Holstein cattle
Source: BMC Genet. 2013 Jun 7;14:49. doi: 10.1186/1471-2156-14-49 (PMC3686577; doi:10.1186/1471-2156-14-49)
Supplement: Additional file 2: Table S4 — Correlations among predicted transmitting ability for traits on bulls used for genotyping. Table S5. Correlations, within DPR class, among predicted transmitting ability for traits on bulls used for genotyping. Table S6. Information for all SNPs with minor allele frequencies (MAF) > 5% and call rates > 70%. [file 1471-2156-14-49-S2.pdf]

Table S4. Correlations among predicted transmitting ability for traits on bulls used for genotyping.[a]

	DPR	HCR	CCR	PL	NM	Milk	FY	FPC	PY	PPC	SCS
HCR	0.61										
	(< 0.0001)										
CCR	0.91	0.69									
	(< 0.0001)	(< 0.0001)									
PL	0.81	0.52	0.75								
	(< 0.0001)	(< 0.0001)	(< 0.0001)								
NM	0.49	0.33	0.45	0.83							
	(< 0.0001)	(< 0.0001)	(< 0.0001)	(< 0.0001)							
MY	-0.45	-0.18	-0.38	-0.04	0.42						
	(< 0.0001)	(< 0.0001)	(< 0.0001)	(0.3104)	(< 0.0001)						
FY	-0.35	-0.17	-0.31	0.06	0.55	0.74					
	(< 0.0001)	(< 0.0001)	(< 0.0001)	(0.1568)	(< 0.0001)	(< 0.0001)					
FPC	0.16	0.03	0.11	0.13	0.14	-0.42	0.30				
	(0.0002)	(0.4158)	(0.0077)	(0.0019)	(0.0007)	(< 0.0001)	(< 0.0001)				
PY	-0.34	-0.15	-0.30	0.07	0.57	0.93	0.83	-0.19			
	(< 0.0001)	(0.0006)	(< 0.0001)	(0.0904)	(< 0.0001)	(< 0.0001)	(< 0.0001)	(< 0.0001)			
PPC	0.31	0.12	0.24	0.28	0.30	-0.33	0.12	0.64	0.04		
	(< 0.0001)	(0.0061)	(< 0.0001)	(< 0.0001)	(< 0.0001)	(< 0.0001)	(0.0035)	(< 0.0001)	(0.298)		
SCS	-0.55	-0.24	-0.50	-0.66	-0.56	0.15	0.04	-0.15	0.08	-0.20	
	(< 0.0001)	(< 0.0001)	(< 0.0001)	(< 0.0001)	(< 0.0001)	(0.0004)	(0.3787)	(0.0003)	(0.0748)	(< 0.0001)	
BY	-0.15	-0.01	-0.14	0.15	0.52	0.63	0.62	-0.05	0.70	0.10	-0.04
	(0.0004)	(0.7883)	(0.0014)	(0.0006)	(< 0.0001)	(< 0.0001)	(< 0.0001)	(0.275)	(< 0.0001)	(0.0201)	(0.4066)

[a]Abbreviations are as follows: DPR, daughter pregnancy rate; HCR, heifer conception rate; CCR, cow conception rate, PL, productive life; NM, net merit; MY, milk yield; FY, fat yield, FPC, fat percent, PY, protein yield; PPC, protein percent; SCS, somatic cell score; BY, birth year. [b]The *P* value of each correlation is in parentheses.

Table S5. Correlations, within DPR class, among predicted transmitting ability for traits on bulls used for genotyping.[ab]

	DPRC	DPR	HCR	CCR	PL	NM	MY	FY	FPC	PY	PPC	SCS
HCR	High	**0.18**										
	low	0.06										
CCR	High	**0.30**	**0.53**									
	low	**0.42**	**0.32**									
PL	High	-0.01	0.10	**0.14**								
	low	**0.55**	0.05	**0.20**								
NM	High	**-0.19**	0.04	0.03	**0.89**							
	low	**0.43**	0.08	0.10	**0.77**							
MY	High	**-0.31**	0.02	0.04	**0.72**	**0.86**						
	low	0.00	**0.22**	-0.05	**0.18**	**0.61**						
FY	High	**-0.30**	-0.01	-0.06	**0.66**	**0.89**	**0.75**					
	low	0.11	**0.14**	0.04	**0.38**	**0.79**	**0.57**					
FPC	High	0.01	-0.05	**-0.14**	-0.08	0.06	**-0.33**	**0.37**				
	low	0.11	-0.10	0.10	**0.20**	**0.14**	**-0.52**	**0.40**				
PY	High	**-0.31**	0.02	0.00	**0.73**	**0.93**	**0.94**	**0.85**	-0.10			
	low	0.05	**0.15**	-0.08	**0.23**	**0.71**	**0.89**	**0.69**	**-0.27**			
PPC	High	-0.01	-0.01	-0.10	0.05	**0.21**	**-0.15**	**0.31**	**0.66**	**0.20**		
	low	0.11	**-0.18**	-0.06	0.07	0.11	**-0.39**	**0.15**	**0.59**	0.08		
SCS	High	-0.04	0.00	0.00	**-0.47**	**-0.42**	**-0.18**	**-0.23**	-0.07	**-0.23**	**-0.12**	
	low	**-0.33**	**0.22**	**-0.14**	**-0.51**	**-0.43**	0.03	-0.09	-0.12	0.05	0.04	
BY	High	**-0.21**	0.10	0.02	**0.61**	**0.78**	**0.70**	**0.70**	0.02	**0.77**	**0.22**	**-0.23**
	low	-0.04	0.08	**-0.13**	0.10	**0.47**	**0.52**	**0.46**	-0.10	**0.59**	0.05	0.00

[a]Abbreviations are as follows: DPRC, daughter pregnancy rate class; DPR, daughter pregnancy rate; HCR, heifer conception rate; CCR, cow conception rate, PL, productive life; NM, net merit; MY, milk yield; FY, fat yield, FPC, fat percent, PY, protein yield; PPC, protein percent; SCS, somatic cell score; BY, birth year. [b]Bold numbers represent significant correlations (*P* < 0.05).

Table S6. Information for all SNPs with minor allele frequencies (MAF) > 5% and call rates > 70%.[ab]

SNP	Gene symbol	Type	Chrom	Location	Alleles and flanking sequence	Call rate (%)	Genotype	Genotype freq (%)	MAF (%)	HWE	Reference
rs109967779	*ACAT2*	M	9	97478396	CTCACTTGCCATTGT[T/C]TGGCCACATTTTCAG	97.09	C/C	15.54	37.17	0.09	27
							C/T	43.26			
							T/T	41.20			
rs133700190	*AP3B1*	M	10	8878196	ATCATGTCATACCTA[T/C]TGCTACGAAGTAAAC	95.27	T/T	4.58	20.52	0.60	40
							T/C	31.87			
							C/C	63.55			
rs41766835	*APBB1*	M	15	45979436	CCTGGCCTGATCATC[A/G]GCACCCAAGAGCAGG	92.00	G/G	4.74	19.47	0.17	38
							G/A	29.45			
							A/A	65.81			
rs110541595	*ARL6IP1*	N	25	17497785	CTCTACTTAAATAAG[A/T]CAAAACCAAAAAAGT	90.36	A/A	16.90	40.04	0.42	38
							A/T	46.28			
							T/T	36.82			
rs110127056	*ASL*	M	25	29655489	GCTCCACGCTGTGTC[C/G]GTAGTCCCACACGTG	96.55	G/G	21.66	45.29	0.29	38
							G/C	47.27			
							C/C	31.07			
rs110909003	*ATP5A1*	M	24	47453621	CATACCTGATTTTGC[T/C]CAACAGGGCCTGATG	70.73	T/T	25.19	48.20	0.12	27
							T/C	46.02			
							C/C	28.79			
rs43114141	*AVP*	M	13	52663154	GAGTGCCGGGAAGGT[A/G]TCGGCTTCCCCCGCC	88.18	A/A	24.54	44.54	< 0.0001	
							A/G	40.00			
							G/G	35.46			
rs109669573	*BCAS1*	M	13	82536290	GCTTGTTGGTCTCCG[T/C]GGCTGCCTTTTTGTC	95.82	T/T	18.79	42.13	0.33	38
							T/C	46.68			
							C/C	34.54			
rs133674837	*BDH2*	M	6	23368958	CACCGATATCAATGA[A/C]TCCAAACTTCAGGAA	98.18	A/A	15.56	38.61	0.53	46
							A/C	46.11			
							C/C	38.33			
rs109032590	*BOLA-DMB*	M	23	7456451	CTCCGTTGGGCTGGA[T/C]GATCCTCCAGGCCCT	95.09	T/T	10.90	29.92	0.03	38
							C/T	38.05			
							C/C	51.05			

Table S6 (continued).[ab]

SNP	Gene symbol	Type	Chrom	Location	Alleles and flanking sequence	Call rate (%)	Genotype	Genotype freq (%)	MAF (%)	HWE	Reference
rs110217852	*BSP3*	M	18	51328727	GGAGTACATTTATGA[A/G]CTATTGGTGCTCACT	97.09	A/A	9.93	30.34	0.43	26
							A/G	40.82			
							G/G	49.25			
rs133455683	*C17H22orf25*	M	17	76444097	TACGGAAACCGGGGG[A/G]AGCGGGAGCCTGTTG	92.18	A/A	19.53	41.12	0.02	32
							A/G	43.20			
							G/G	37.28			
rs135390325	*C1QB*	M	2	134538911	CCCGGGCACCTTGCAC[A/G]TGAACTTGCTGCTCC	98.36	A/A	0.37	9.61	0.14	38
							A/G	18.48			
							G/G	81.15			
rs134150850	*C28H10ORF10*	M	28	44812770	CTCTGCAGAGAACAT[T/C]GAGGGACTGGCACCA	94.91	C/C	18.58	42.72	0.76	38
							C/T	48.28			
							T/T	33.14			
rs109332658	*C7H19orf60*	M	7	4529011	CACACCAGCTCCTCG[T/C]GCTCTGGGACTCCAG	93.64	T/T	7.57	27.18	0.83	38
							T/C	39.22			
							C/C	53.20			
rs135744058	*CACNA1D*	M	22	48255786	GGTAGCCTCGAGGCC[A/G]CTCAAAGTCCAAGCT	98.55	G/G	6.27	25.74	0.67	25
							G/A	38.93			
							A/A	54.80			
rs42940189	*CALCR*	M	4	11008943	TGTACCAAACCCCAT[T/C]TTCCTCACAATACTT	84.73	T/T	1.07	9.23	0.57	25
							T/C	16.31			
							C/C	82.62			
rs137601357	*CAST*	M	7	97350820	CTGGTGAAAAAGCCC[T/C]GGTCCCCAAGGTCAG	99.09	C/C	18.53	42.84	0.87	15
							C/T	48.62			
							T/T	32.84			
rs109405883	*CCDC137*	M	19	52643332	GGACATAGGCCCTGT[T/C]GGACTCCCACTTCCT	92.00	T/T	23.91	48.72	0.87	25
							T/C	49.60			
							C/C	26.48			
rs109447102	*CCDC86*	M	29	38918350	CCGGTCAGAAAGCAC[T/C]GGGTCCCGAGCCCTC	95.27	C/C	4.20	18.42	0.22	32
							C/T	28.44			
							T/T	67.37			

Table S6 (continued).[ab]

SNP	Gene symbol	Type	Chrom	Location	Alleles and flanking sequence	Call rate (%)	Genotype	Genotype freq (%)	MAF (%)	HWE	Reference
rs41652321	*CCDC88B*	M	29	44427483	GAAGACTCGGTGATT[C/G]GAGAGCCGGACTTAG	96.36	G/G	1.51	8.58	0.02	25
							G/C	14.15			
							C/C	84.34			
rs137673698	*CCT8*	M	1	6426316	ACTTACCTGGGAAAA[T/C]ACTGGGCTATTAAAC	97.64	T/T	0.19	46.93	< 0.0001	27
							T/C	93.48			
							C/C	6.33			
rs109621328	*CD14*	M	7	51051802	GTGCTTGGGCAATGT[T/C]CAGCACCCTGAGCCC	98.36	C/C	1.11	7.58	0.08	35
							C/T	12.94			
							T/T	85.95			
rs133747802	*CD2*	M	3	28824448	CACACGGCGATCTCC[A/G]TGCTGACTTTCTTAT	80.91	G/G	34.16	40.22	< 0.0001	32
							G/A	12.13			
							A/A	53.71			
rs41711496	*CD40*	M	13	75633238	TCGACACATACACAA[A/G]TGGTGTCTGTATTCA	97.27	A/A	26.36	49.72	0.13	38
							A/G	46.73			
							G/G	26.92			
rs41857027	*CFDP2*	M	18	2160205	CGGCGCCTTTTTCCT[T/G]CTCTGCAGCTGCATC	86.91	T/T	8.58	17.15	< 0.0001	34
							T/G	17.15			
							G/G	74.27			
rs109301586	*COQ9*	M	18	25217841	TTGGATCAGCAGAAG[A/G]ATCAACCCCCGCCCT	92.91	G/G	28.96	48.53	< 0.0001	33
							G/A	39.14			
							A/A	31.90			
rs134432442	*CPSF1*	M	14	1736599	AGCGTGTGGACGCCA[T/C]CACGGGCTGGTCAGG	95.27	T/T	5.73	22.71	0.46	37
							T/C	33.97			
							C/C	60.31			
rs133449166	*CSNK1E*	M	5	116920083	CCAGGGAGAAGGAAG[A/C]CACAATGAAGGGGTC	94.73	C/C	17.47	40.60	0.35	34
							C/A	46.26			
							A/A	36.28			
rs109443582	*CSPP1*	M	14	31281509	CACCGGGGTGACGAC[A/G]GATCAAACTCTGTAG	90.00	A/A	0.40	6.87	0.81	38
							A/G	12.93			
							G/G	86.67			

Table S6 (continued).[ab]

SNP	Gene symbol	Type	Chrom	Location	Alleles and flanking sequence	Call rate (%)	Genotype	Genotype freq (%)	MAF (%)	HWE	Reference
rs110270752	*DEPDC7*	M	15	63421395	TAATGGATCATCAAA[A/G]AGATGTTTTTAAGGT	94.18	A/A	5.79	20.95	0.05	33
							A/G	30.31			
							G/G	63.90			
rs110629231	*DNAH11*	M	4	31908360	CTGAGTCTGCATCTA[T/C]ACCCAGCGAGCATGT	98.00	T/T	11.69	32.93	0.38	25
							T/C	42.49			
							C/C	45.83			
rs109455239	*DOK2*	M	8	72427041	GGGACAGTGGGCGAC[A/G]GTGGTGGCAGCGAGT	95.09	A/A	4.40	20.27	0.68	25
							A/G	31.74			
							G/G	63.86			
rs109503725	*DSC2*	M	24	27124361	CCAGTCATACGTCAG[T/C]GGACGGTGGTCATCT	97.27	C/C	26.54	49.63	0.08	29
							C/T	46.17			
							T/T	27.29			
rs42075611	*DTX2*	M	25	36615779	CATCATCTGTATGGA[C/G]AAGCTGTCCGTGGCG	87.82	C/C	3.11	7.35	< 0.0001	31
							C/G	8.49			
							G/G	88.41			
rs109561866	*DYRK3*	M	16	3449362	GCAAAGATTGGGTGA[T/C]GGCACTGAAAGGGTG	96.18	T/T	1.13	11.15	0.80	33
							T/C	20.04			
							C/C	78.83			
rs133175991	*DZIP3*	M	1	54924494	TCAGTTTCTCCATAT[T/C]CTGTAGTTATCTTGG	87.64	C/C	3.73	19.29	0.99	32
							C/T	31.12			
							T/T	65.15			
rs43676052	*EPAS1*	M	11	29886509	GGCCAAGCAGCTCTT[C/G]AGGGTGGTAACCAAC	97.64	G/G	3.54	21.69	0.11	38
							G/C	36.31			
							C/C	60.15			
rs135071345	*FAM5C*	M	16	14213274	GCGCCAATAGCTCCT[T/C]GGTTACTCTGGAGAC	98.55	C/C	2.21	15.96	0.56	25
							C/T	27.49			
							T/T	70.30			
rs109137982	*FCER1G*	M	3	9216708	CTTGTTCAACCAAAA[A/G]GAGTAAGAGCAGGAC	98.91	A/A	0.55	9.65	0.31	38
							A/G	18.20			
							G/G	81.25			

Table S6 (continued).[ab]

SNP	Gene symbol	Type	Chrom	Location	Alleles and flanking sequence	Call rate (%)	Genotype	Genotype freq (%)	MAF (%)	HWE	Reference
FGF2ag	*FGF2*	I	17	1186	GTTAGGGCCCACTCT[A/G]TAACAGCCCCATTTT	99.45	A/A	14.08	35.56	0.14	16
							A/G	42.96			
							G/G	42.96			
rs43745234	*FSHR*	M	11	32613008	CTGCTATACATCGAC[C/G]CTGATGCCTTCCAGA	98.91	G/G	8.64	30.42	0.50	
							G/C	43.57			
							C/C	47.79			
rs109247499	*FST*	M	20	27301549	TGCGGTGAGCTGTGC[C/G]CTGAGAGTAAGTCTG	96.91	C/C	26.27	49.81	0.18	43
							C/G	47.09			
							G/G	26.64			
rs41893756	*FUT1*	M	18	55205427	CTACGGTCTGGACAC[A/G]GCAGGAACAGGTCTA	96.36	G/G	5.47	19.43	0.01	30
							G/A	27.92			
							A/A	66.60			
rs109262355	*FYB*	M	20	37482030	CCTCTCTGTGGAGCC[A/G]CAGTCTTGGTGGGGC	97.27	G/G	15.14	37.20	0.20	32
							G/A	44.11			
							A/A	40.75			
rs109830880	*GCNT3*	M	10	51100846	GTGATCCCAGAACAG[T/C]TGATGGATCTCTTTG	75.45	C/C	1.69	13.01	0.99	33
							C/T	22.65			
							T/T	75.66			
rs42339105	*GOLGA4*	M	22	10888644	TTAAGTTGGGATTCA[T/G]AGAGCTGGGTTAAAG	90.91	G/G	0.40	8.90	0.28	30
							G/T	17.00			
							T/T	82.60			
rs109516714	*GPLD1*	M	23	33332090	TTGGAGAGGAGTATA[T/C]GGGAGATGTTTATTG	95.09	C/C	16.44	39.87	0.60	38
							C/T	46.85			
							T/T	36.71			
rs110733329	*HAX1*	M	3	17635327	GGAGTCACGTGGGGC[T/C]GTGGGAACTCGAGGT	98.36	T/T	0.55	6.75	0.71	38
							T/C	12.38			
							C/C	87.06			
rs109711583	*HSD17B12*	M	15	74233184	AAACTGGCTAAAATC[A/G]GAAAGCCAACTTTGG	98.18	G/G	19.26	43.98	0.94	
							G/A	49.44			
							A/A	31.30			

Table S6 (continued).[ab]

SNP	Gene symbol	Type	Chrom	Location	Alleles and flanking sequence	Call rate (%)	Genotype	Genotype freq (%)	MAF (%)	HWE	Reference
rs43079452	*HSD17B3*	M	8	87190501	TCTTTCTTGAAGTCA[A/G]TGGGAGAATGGGCAG	98.36	G/G	1.85	14.23	0.74	
							G/A	24.77			
							A/A	73.38			
rs109769865	*HSD17B6*	M	5	61129370	ACCACCTGCCTCTCT[T/C]GGTACCAGCGCAGGA	96.55	T/T	3.01	14.03	0.05	
							T/C	22.03			
							C/C	74.95			
rs110828053	*HSD17B7*	M	3	7226375	AAAATTTTTCAGCAG[T/C]GTCTTCATCTAGGTC	98.73	T/T	4.24	19.43	0.49	
							T/C	30.39			
							C/C	65.38			
HSP70C895D	*HSPA1A*	P	23	Unknown	TTGGTTCCAGAAAGC[-/C]AGGGGGCAGGACTTG	99.45	-/-	8.41	28.24	0.62	23
							-/C	39.67			
							C/C	51.92			
rs110789098	*IBSP*	M	6	37683555	CATACTCCCCAGGGG[T/C]GGTGATTTTCCCAAA	93.82	C/C	22.09	43.80	0.01	32
							C/T	43.41			
							T/T	34.50			
rs133621844	*IFNT2*	M	8	23638202	TGTAGAAGAGGTTGA[A/G]GCACTGCTGGAGCAT	95.64	A/A	0.57	12.45	0.04	
							A/G	23.76			
							G/G	75.67			
rs110953315	*IRF9*	M	10	21270470	CACTTTGGGAAGAGT[A/G]AGACCCTGGCCCTTG	98.55	A/A	2.21	14.67	0.91	28
							A/G	24.91			
							G/G	72.88			
rs111015912	*LDB3*	M	28	41302317	AGAACCAGAACAAGC[T/C]GGAAGATGAGGCTGAT	96.00	T/T	4.73	22.25	0.77	40
							T/C	35.04			
							C/C	60.23			
rs41256848	*LHCGR*	M	11	32192962	CACACTAGAAAGATG[T/G]CACACCATCACCTAT	99.27	T/T	17.03	42.31	0.41	
							T/G	50.55			
							G/G	32.42			
rs43362191	*MACF1*	M	3	113759645	ATGCGTTGTCTTGCC[A/G]AGCATCTTTGTCAGT	97.45	A/A	18.84	41.88	0.22	38
							A/G	46.08			
							G/G	35.07			

Table S6 (continued).[ab]

SNP	Gene symbol	Type	Chrom	Location	Alleles and flanking sequence	Call rate (%)	Genotype	Genotype freq (%)	MAF (%)	HWE	Reference
rs134011564	*MARVELD1*	M	26	20538410	CTTGGTACCGGCTGG[T/G]GGCGATGGTGATCCA	78.36	G/G	0.46	42.81	< 0.0001	38
							G/T	84.69			
							T/T	14.85			
rs41859871	*MON1B*	M	18	3302068	AACGAATGAAGAGCC[A/G]ATCCTCCTCTTTCTT	99.27	A/A	1.10	11.45	0.63	33
							A/G	20.70			
							G/G	78.21			
rs109248655	*MRGPRF*	M	29	48282900	CAGGAAGATGTAGTT[T/C]ATGATGGCCGGCGGC	97.45	T/T	0.00	6.62	0.35	38
							T/C	13.06			
							C/C	86.94			
rs43703916	*MRPL48*	M	15	53053970	CTTTTGCAGATCAGC[A/G]GTTTGAATGCTACGT	91.82	A/A	24.16	49.21	0.96	38
							A/G	50.10			
							G/G	25.74			
rs109761676	*MS4A8B*	M	29	38810808	CCTACATCTACCCTT[T/G]GTATGATCCTCCCTC	94.36	G/G	6.74	27.36	0.39	31
							G/T	41.23			
							T/T	52.02			
rs133762601	*NEU3*	M	15	54014799	GCCCCCTCATGGCTG[T/G]CAAGGCAGTGTGGTG	92.55	G/G	15.32	18.27	< 0.0001	35
							G/T	5.89			
							T/T	78.78			
rs133497176	*NFKBIL1*	M	23	27512896	CTTGGGGGGAGGGGT[T/C]ACTCATCAGACCTGC	96.55	T/T	2.64	13.56	0.12	38
							T/C	21.85			
							C/C	75.52			
rs109383758	*NLRP9*	M	18	62170785	CTTCACAGAGGTCAG[T/C]GTTGCAATCCAAGTC	99.45	C/C	25.78	49.63	0.29	21
							C/T	47.71			
							T/T	26.51			
rs42508588	*NT5E*	M	9	65766876	AGGATCACCTTGTAC[A/T]CCTTATCCATTCTAA	97.82	T/T	1.67	16.45	0.08	33
							T/A	29.55			
							A/A	68.77			
rs134264563	*OCLN*	M	20	10830360	GAAGACTCTGGATAC[A/G]GTCGCTTCTCGTTCA	98.73	G/G	7.92	29.01	0.58	27
							G/A	42.17			
							A/A	49.91			

Table S6 (continued).[ab]

SNP	Gene symbol	Type	Chrom	Location	Alleles and flanking sequence	Call rate (%)	Genotype	Genotype freq (%)	MAF (%)	HWE	Reference
rs111027720	*PARM1*	M	6	93134487	TCACCTCCCCTCAAG[C/G]GCCAGCCTCATCGCC	97.09	C/C	22.28	45.79	0.22	38
							C/G	47.00			
							G/G	30.71			
rs109813896	*PCCB*	M	1	135150532	AGGCTCAGTGTTGTA[C/G]TGAGAAGTCTCCGGG	98.00	C/C	13.36	34.97	0.25	46
							C/G	43.23			
							G/G	43.41			
rs109506766	*PGR*	I	15	6576069	TAATGGTGATCTAAA[C/G]ACATGGTGATCTGCT	99.45	G/G	13.53	36.47	0.82	14
							G/C	45.89			
							C/C	40.59			
rs41749178	*PLET1*	M	15	20989944	GGCTCCTGGGAAAAC[T/C]CCAGCGACCATTGTG	96.36	C/C	5.85	22.36	0.26	38
							C/T	33.02			
							T/T	61.13			
rs109629628	*PMM2*	M	25	8565782	TCGTGGAGGATCTGC[A/G]AAAAGAGTTTGCTGG	95.64	G/G	14.26	38.02	0.85	32
							G/A	47.53			
							A/A	38.21			
rs133729105	*RABEP2*	M	25	27716726	AAGGACCTGGAGAGC[A/G]TCAGCCGGGAGCGGG	96.18	A/A	15.69	37.43	0.10	38
							A/G	43.48			
							G/G	40.83			
rs135078185	*RALGPS1*	M	11	101468911	TCTGTGGCCGGCAGC[A/C]TCCCCACACCCCCGG	82.00	A/A	30.16	30.49	< 0.0001	32
							A/C	0.67			
							C/C	69.18			
rs43572154	*ROR2*	M	8	90424655	CGGGGCTCTGGGCCA[T/C]GTCTTCCGGGACATT	99.45	T/T	1.83	15.08	0.41	32
							T/C	26.51			
							C/C	71.66			
rs136457441	*RPL26*	M	19	28520752	TGGACTACTTTGCCA[A/G]TTTGCTGCCCTTTGT	91.82	G/G	22.18	48.42	0.26	48
							G/A	52.48			
							A/A	25.35			
rs136746215	*SEC14L1*	M	19	56362555	CTGCCCCACTCGGGG[T/G]GCTGAGGGGCTCGCC	72.36	G/G	19.66	34.42	< 0.0001	38
							G/T	43.26			
							T/T	37.08			

Table S6 (continued).[ab]

SNP	Gene symbol	Type	Chrom	Location	Alleles and flanking sequence	Call rate (%)	Genotype	Genotype freq (%)	MAF (%)	HWE	Reference
rs43321188	*SERPINE2*	M	2	116547485	GTAGCATCCCCAGGA[T/C]GGACGCAATCCCATG	97.82	T/T	7.99	26.21	0.18	42
							T/C	36.43			
							C/C	55.58			
rs110365063	*SLC18A2*	M	26	38071106	CTAGAAATCCAGACC[A/G]CCAAGCCCGGGCTCA	97.82	A/A	2.42	15.43	0.95	39
							A/G	26.02			
							G/G	71.56			
rs41912290	*SREBF1*	M	19	35684696	ACGTGGCCGGAGCTC[T/C]TACCTGCAGCTTCTC	94.36	C/C	19.85	41.71	0.02	46
							C/T	43.74			
							T/T	36.42			
rs137182814	*STAT5A*	S	19	43740886	CTCAGCCCTGGTGAC[C/G]AGGTGACTCCTGGCC	97.09	G/G	19.66	41.29	0.01	12
							G/C	43.26			
							C/C	37.08			
rs42158454	*SYTL2*	M	29	10194073	ACCAATCTTAGCAGC[A/T]CCTCTGGCATGACGT	96.00	A/A	0.00	8.33	0.04	25
							A/T	16.67			
							T/T	83.33			
rs42525506	*TAF9*	M	20	10986894	GCACACCAACCCCAC[A/C]AGCCATGTCTGTTTC	80.00	A/A	9.09	46.02	< 0.0001	36
							A/C	73.86			
							C/C	17.05			
rs110660625	*TBC1D24*	N	25	2514825	CGTGGCTGGTGACTG[A/G]CGCTAAAATGCCTCC	96.18	A/A	12.10	33.36	0.32	32
							A/G	42.53			
							G/G	45.37			
rs110805802	*TDRKH*	M	3	20519342	AGGAAGAGAATTGCT[T/C]ATTCTGCAGAAACCA	92.91	T/T	1.96	12.82	0.53	33
							T/C	21.72			
							C/C	76.32			
rs132789482	*TSHB*	M	3	30778061	AATATCCAGCACAGA[T/C]GGTGGTGTTGATGGT	75.45	T/T	2.89	14.10	0.13	
							T/C	22.41			
							C/C	74.70			
rs43445397	*TSPYL1*	M	9	36151521	AATGTCGACGCATCC[A/G]CCCGAATTTATGCT	99.45	A/A	0.00	49.91	< 0.0001	25
							A/G	99.82			
							G/G	0.18			

Table S6 (continued).[ab]

SNP	Gene symbol	Type	Chrom	Location	Alleles and flanking sequence	Call rate (%)	Genotype	Genotype freq (%)	MAF (%)	HWE	Reference
rs135236119	*TTLL3*	M	22	17320731	TGCGGTCCAGTCGCC[A/G]GTCGATGACCACACG	95.64	A/A	6.46	23.67	0.27	32
							A/G	34.41			
							G/G	59.13			
rs134031231	*TXN2*	M	5	80313306	TGTACTGTGGGGTCT[T/G]CAGGGCCCTGGAGGC	96.36	G/G	18.30	43.02	0.85	35
							G/T	49.43			
							T/T	32.26			
rs134818016	*UHRF1*	F	7	17836918	CTGCGAGGACGATGT[-/G]GGCCGACGCCATCTT	85.64	G/G	2.76	8.28	< 0.0001	34
							G/-	11.04			
							-/-	86.20			
rs137248155	*VCAN*	M	7	84362267	TCACAGAGAAAAATC[T/C]TGAACTTGATCATCT	97.82	T/T	9.11	28.72	0.33	42
							T/C	39.22			
							C/C	51.67			
rs134282928	*WBP1*	M	11	10478345	TCGAGGGGCAGCCAG[T/G]GGCTGCAGTGTAAGG	91.45	G/G	0.60	13.52	0.02	38
							G/T	25.84			
							T/T	73.56			
rs43332935	*WDR77*	M	3	34445120	AGTGCAGGCTGTGCC[C/G]TCAGCTCTGTTGTGC	99.09	G/G	0.92	10.64	0.60	44
							G/C	19.45			
							C/C	79.63			
rs43662395	*ZNF638*	M	11	13766506	TCAATCATGGTTTTC[A/G]GATTTTCTTCCATAA	91.82	A/A	0.59	5.64	0.24	31
							A/G	10.10			
							G/G	89.31			
rs110883602	*ZP2*	M	25	20183725	GGTTTTGTTTGATGG[T/C]GGTAGACCTCGACGT	74.91	C/C	20.15	39.44	< 0.0001	37
							C/T	38.59			
							T/T	41.26			

[a]Single nucleotide polymorphism represented as the rs number designated by the National Center for Biotechnology Information dbSNP. For entries not beginning with rs, the abbreviation given by previous researchers was used. [b]Abbreviations are: SNP, single nucleotide polymorphism; Type, type of polymorphism; F, frameshift; I, intron; N, nonsense; M, missense; P, promoter; S, synonymous; Chrom, chromosome in which SNP was located; Location, location within the chromosome which the SNP was located; Genotype freq, genotype frequency; MAF, minor allele frequency; HWE, probability of SNP being in Hardy Weinberg Equilibrium; Reference, reference from which the gene was obtained (SNPs without references were chosen due to having a well-known role in reproductive processes).
