# Supplementary material for: Discovery of single nucleotide polymorphisms in candidate genes associated with fertility and production traits in Holstein cattle
Source: BMC Genet. 2013 Jun 7;14:49. doi: 10.1186/1471-2156-14-49 (PMC3686577; doi:10.1186/1471-2156-14-49)
Supplement: Additional file 3: Table S7 — Genetic standard deviations of SNPs associated with daughter pregnancy rate. Table S8. Effect of tissue type in which genes were identified on the percent of genes that were significantly associated with daughter pregnancy rate (DPR). Figure S1. The ubiquitin pathway contains an overrepresentation of daughter pregnancy rate genes. Figure S2. Transcription factors which regulate daughter pregnancy rate genes. [file 1471-2156-14-49-S3.pdf]

Table S7. Genetic standard deviations of SNPs associated with daughter pregnancy rate.[a]

SNP	Gene	Chromosome	Location	Marker effect (genetic standard deviation)
rs109813896	*PCCB*	1	134130474	0.67
rs135390325	*C1QB*	2	130769591	0.99
rs110828053	*HSD17B7*	3	6630548	0.73
rs110805802	*TDRKH*	3	19048120	1.78
rs133449166	*CSNK1E*	5	110565337	0.47
rs111027720	*PARM1*	6	91677982	1.52
rs109332658	*C7H19orf60*	7	4533772	0.46
rs109621328	*CD14*	7	53448291	1.09
rs137601357	*CAST*	7	98485273	0.72
rs109967779	*ACAT2*	9	97478396	0.66
rs133700190	*AP3B1*	10	9177305	0.77
rs41711496	*CD40*	13	75567844	0.49
rs134432442	*CPSF1*	14	1736599	0.54
rs109443582	*CSPP1*	14	33342060	1.06
rs41766835	*APBB1*	15	47252371	0.63
rs133762601	*NEU3*	15	55041713	0.55
rs110270752	*DEPDC7*	15	64476283	0.82
rs109711583	*HSD17B12*	15	74828355	0.47
rs109561866	*DYRK3*	16	4284409	0.63
rs133455683	*C17H22orf25*	17	74976374	0.51
rs41857027	*CFDP2*	18	2783606	0.61
rs41859871	*MON1B*	18	4453289	0.99
rs109301586	*COQ9*	18	25527339	0.85
rs110217852	*BSP3*	18	51919757	0.53
rs41893756	*FUT1*	18	55831611	0.97
rs109383758	*NLRP9*	18	62241722	0.44
rs134264563	*OCLN*	20	10167825	0.65
rs135744058	*CACNA1D*	22	47726446	0.68
rs133497176	*NFKBIL1*	23	27560559	0.75
rs109516714	*GPLD1*	23	33016354	0.61
rs109503725	*DSC2*	24	26370779	0.52
rs110660625	*TBC1D24*	25	2007163	0.72
rs109629628	*PMM2*	25	7716425	0.77
rs110883602	*ZP2*	25	19232797	0.93
rs133729105	*RABEP2*	25	26182660	0.62
rs134011564	*MARVELD1*	26	18733146	1.15
rs111015912	*LDB3*	28	41679976	0.99
rs109761676	*MS4A8B*	29	37646668	0.86
rs109447102	*CCDC86*	29	37751619	0.66
rs109248655	*MRGPRF*	29	47011635	0.95

[a]Single nucleotide polymorphism represented as the rs number given by the National Center for Biotechnology Information data base SNP.

Table S8. Effect of tissue type in which genes were identified on the percent of genes that were significantly associated with daughter pregnancy rate (DPR).[a]

**Category**	**Gene symbol**		**Fraction and percent related to DPR**
	**Significant**	**Non-significant**	
Genes expressed in brain or pituitary	*CACNA1D, TSHB*	*AVP, CALCR, CCDC137, CCDC88B, DOK2, DNAH11, FAM5C, SYTL2, TSPYL1*	2/11 (18%)
Genes expressed in the embryo	*ACAT2, C17H22orf25, CCDC86, CD14, CFDP2, CSNK1E, COQ9, DEPDC7, DSC2, DYRK3, FUT1, MON1B, MS4A8B, NEU3, OCLN, PMM2, TBC1D24, TDRKH*	*ATP5A1, CCT8, CD2, DTX2, DZIP3, FYB, GCNT3, GOLGA4, IBSP, NT5E, RALGPS1, ROR2, TAF9,, TTLL3, TXN2, UHRF1, ZNF638*	18/35 (51%)
Genes expressed in the endometrium or oviduct	*APBB1, ARL6IP1, BSP3, C1QB, CD40, CSPP1, GPLD1,MARVELD1, MRGPRF, PARM1, NFKBIL1, RABEP2,*	*ASL, BCAS1, BOLA-DMB, C28H10orf10, EPAS1, FCER1G, HAX1, MACF1, PLET1, RPL26, SEC14L1, WBP1*	12/24 (50%)
Genes differentially expressed in the ovary	*AP3B1, CPSF1, HSD17B12, HSD17B7,LDB3, ZP2*	*FST, HSD17B3, HSD17B6, LHCGR, SERPINE2, SLC18A2, VCAN, WDR77*	6/14 (43%)

[a]Only genes with MAF > 5% and call rate > 70% are shown.

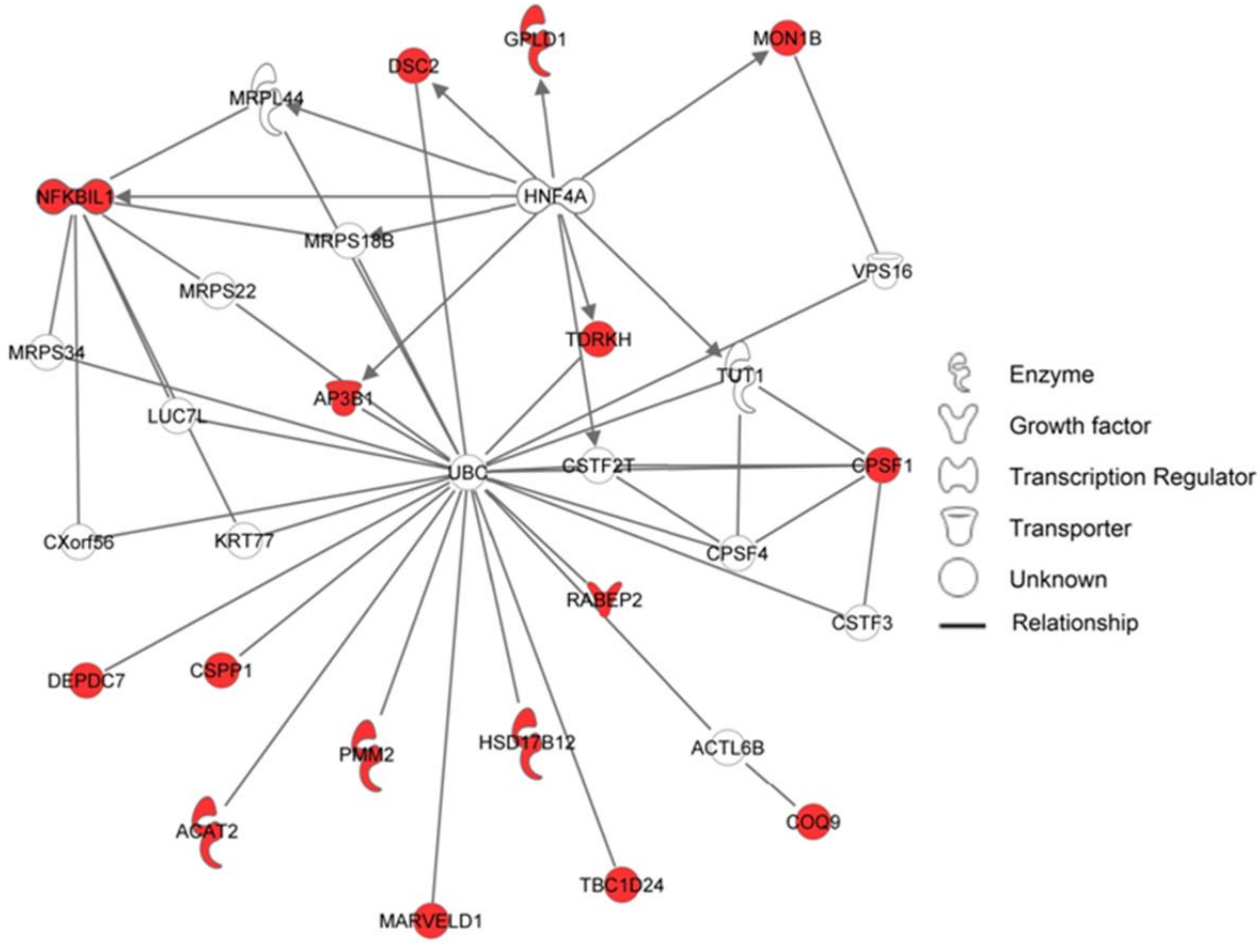

Figure S1 The ubiquitin pathway contains an overrepresentation of daughter pregnancy rate genes. Red symbols are genes containing SNPs associated with daughter pregnancy rate and lines represent a relationship between two genes or their products.

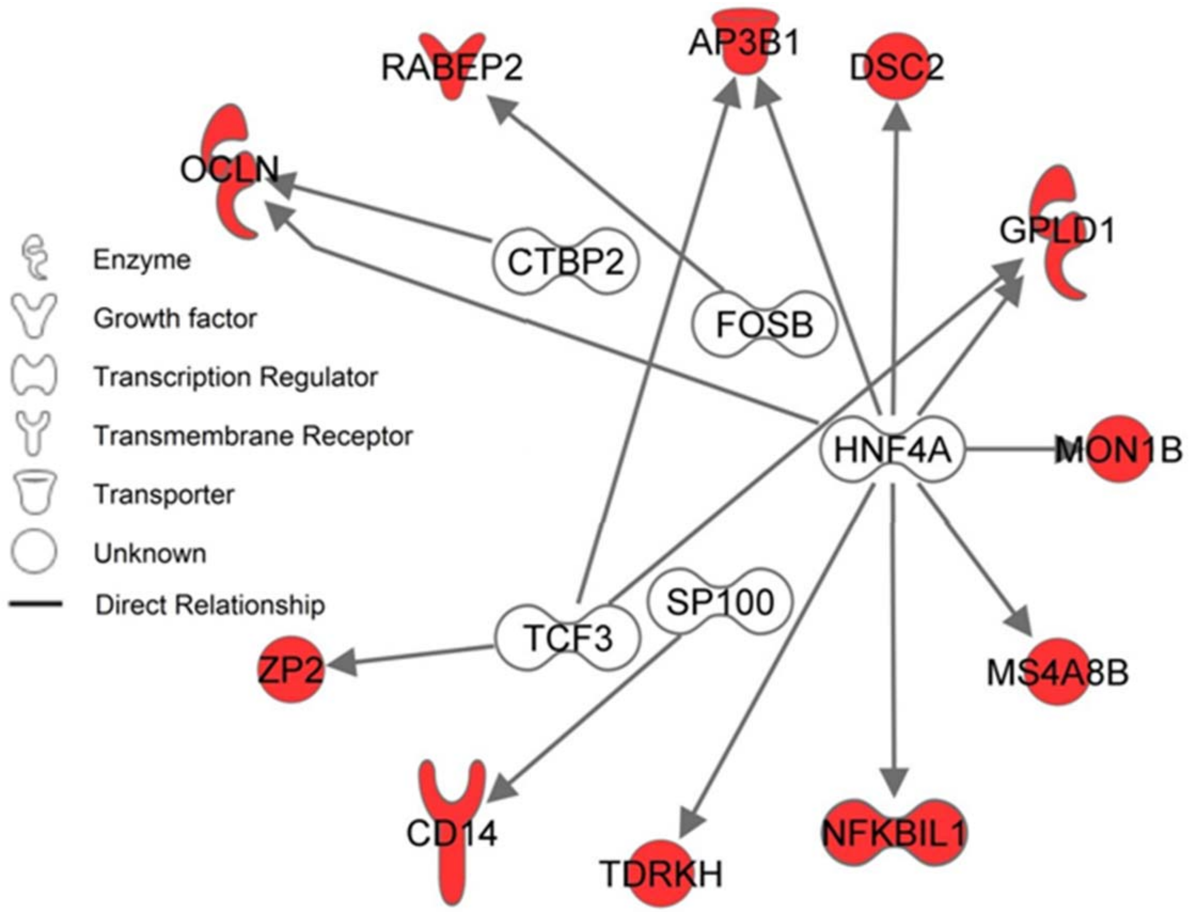

Figure S2 Transcription factors which regulate daughter pregnancy rate genes. Only significant pathways are shown ($P < 0.05$). Red symbols are genes containing SNPs associated with daughter pregnancy rate and arrows represent transcriptional regulation.
